# The Epstein-Barr Virus-Encoded MicroRNA MiR-BART9 Promotes Tumor Metastasis by Targeting E-Cadherin in Nasopharyngeal Carcinoma

**DOI:** 10.1371/journal.ppat.1003974

**Published:** 2014-02-27

**Authors:** Chung-Yuan Hsu, Yung-Hsiang Yi, Kai-Ping Chang, Yu-Sun Chang, Shu-Jen Chen, Hua-Chien Chen

**Affiliations:** 1 Graduate Institute of Biomedical Sciences, College of Medicine, Chang Gung University, Taoyuan, Taiwan, Republic of China; 2 Molecular Medicine Research Center, Chang Gung University, Taoyuan, Taiwan, Republic of China; 3 Department of Otolaryngology, Chang Gung Memorial Hospital at Lin-Kou, Taoyuan, Taiwan, Republic of China; 4 Department of Biomedical Sciences, College of Medicine, Chang Gung University, Taoyuan, Taiwan, Republic of China; University of North Carolina at Chapel Hill, United States of America

## Abstract

MicroRNAs (miRNAs) are a family of small RNA molecules that negatively regulate the expression of protein-coding genes and play critical roles in orchestrating diverse cellular processes. This regulatory mechanism is also exploited by viruses to direct their life cycle and evade the host immune system. Epstein-Barr virus (EBV) is an oncogenic virus that is closely associated with multiple human diseases, including nasopharyngeal carcinoma (NPC), which is a highly metastatic type of tumor and is frequently reported in South Asia. Several viral proteins have been found to promote the migration and invasiveness of NPC cells. However, not all tumor tissues express these viral oncoproteins, suggesting that other mechanisms may contribute to the aggressive behavior of NPC tumor cells. A previous sequencing study by our group revealed that the EBV miRNA miR-BART9 was expressed at high levels in all EBV-positive NPC tissues. In the present study, we used gain- and loss-of-function approaches to investigate the effect of miR-BART9 in EBV-negative and EBV-positive NPC cells. We discovered that miR-BART9 promotes the migration and invasiveness of cultured NPC cells. The promigratory activity observed in vitro was manifested as an enhanced metastatic ability *in vivo*. Computational analysis revealed that miR-BART9 may target E-cadherin, a membrane protein that is pivotal in preserving cell-cell junctions and the epithelial phenotype. Through biochemical assays and functional rescue analysis, we confirmed that miR-BART9 specifically inhibits E-cadherin to induce a mesenchymal-like phenotype and promote the migration of NPC cells. These results indicated that miR-BART9 is a prometastatic viral miRNA and suggested that high levels of miR-BART9 in EBV-positive NPC cells may contribute to the aggressiveness of tumor cells.

## Author Summary

MicroRNAs (miRNAs) are a family of small RNA molecules that negatively regulate the expression of protein-coding genes and orchestrate diverse cellular processes. This regulatory mechanism is also exploited by viruses to manage their life cycle and to evade the host immune system. Epstein-Barr virus (EBV) is closely associated with nasopharyngeal carcinoma (NPC), which is a highly metastatic type of tumor. A previous by our group study discovered that the EBV miRNA miR-BART9 is expressed at high levels in all EBV-positive NPC tissues. In the present study, we used gain- and loss-of-function approaches to investigate the function of miR-BART9 in EBV-negative and EBV-positive NPC cells. We showed that miR-BART9 promotes the migration and invasiveness of cultured NPC cells and enhances the metastatic ability of NPC tumors *in vivo*. Through computational analysis, we discovered that E-cadherin, a membrane protein that is critical for maintaining cell-cell contact and suppressing tumor metastasis, is a potential target for miR-BART9. Through biochemical assays and a functional rescue analysis, we confirmed that miR-BART9 specifically inhibits E-cadherin to enhance the motility of NPC cells. Our results show that miR-BART9 is a prometastatic viral miRNA and suggest that miR-BART9 may contribute to the aggressiveness of EBV-positive NPC tumors.

## Introduction

Epstein-Barr virus (EBV) is an oncogenic virus that is closely tied to several human malignancies, including Burkitt's lymphoma, Hodgkin's disease, extra-nodal nasal natural killer/T cell lymphoma (NKTCL), nasopharyngeal carcinoma (NPC) and gastric cancer [1]–[2]. Products of the EBV genome, such as the EBV-encoded RNAs (EBER), have been detected in nearly all NPC tumor cells, as determined via in situ hybridization [3]–[4]. It is now well recognized that EBV facilitates the pathogenesis of NPC, as several EBV genome products have been shown to participate in the multi-step development of the malignancy. Several EBV viral proteins, including LMP1, LMP2A and EBNA1, possess transforming activity and can stimulate the growth, survival and invasion capability of transformed cells [5]–[8].

In addition, EBV was the first human virus found to encode microRNAs (miRNAs) [9]. MiRNAs are a family of small regulatory RNA molecules involved in a wide variety of cellular processes related to organ development and metabolic homeostasis as well as oncogenic transformation [10]–[12]. MiRNAs suppress the expression of protein-coding genes through imperfect base pairing with the 3′untranslated (3′UTR) region of target messenger RNAs (mRNAs) [11]. To date, a total of 25 EBV miRNA precursors with 44 mature EBV miRNAs have been identified and deposited in the miRBase database (http://microrna.sanger.ac.uk/). These miRNAs are transcribed as 2 clusters in the EBV genome. The BHRF cluster located within the 3′ UTR of the BHRF1 (Bam HI fragment H rightward open reading frame 1) expresses 4 mature miRNAs from 3 precursor-miRNAs. The remaining 40 mature miRNAs are produced from 22 precursor-miRNAs in the BART (Bam HI A region rightward transcript) region [13]. Analysis of EBV-infected cell lines revealed that EBV miRNAs display a cell type-specific expression pattern [14]. BHRF cluster miRNAs are highly expressed in lytic and latency III-infected cell lines, such as B lymphoma cells, but are almost undetectable in cells with latency I and II infections, such as NPC cells. In contrast, BART cluster miRNAs are detected in all types of latent infections as well as lytic infections [14]. Interestingly, BART cluster miRNAs are highly expressed in latently infected epithelial cells but are expressed at a significantly lower levels in B cells [15], suggesting that these miRNAs may contribute to the development of epithelial cancer.

Several EBV miRNAs are known to directly inhibit viral proteins. For example, miR-BART2 suppresses the viral DNA polymerase BALF5 to inhibit EBV lytic replication [16]. miR-BART5-5p, miR-BART19-5p suppress the viral oncoprotein LMP1 and BART10-5p inhibits BHRF1 expression in Jijoye BL cells [17], while miR-BART22 represses LMP2A [18]. Some EBV miRNAs have been found to target cellular protein-coding genes involved in immune response. For example, miR-BHRF1-3 represses the cellular interferon-induced chemokine CXCL11 [19] to modulate the host response during lytic infection, while miR-BART2-5p targets MICB to escape recognition and elimination by NK cells [20]. Similarly, miR-BART15 targets the inflammasome protein NLRP3, leading to the production of IL-1β [21]. Other EBV miRNAs have been shown to target cellular genes involved in growth and survival. For example, miR-BART3* targets the tumor suppressor DICE1 to promote cellular growth and transformation in NPC [22], whereas miR-BART15-3p induces apoptosis by inhibiting the apoptosis inhibitor BRUCE in a gastric cancer cell line [23]. Together, these observations indicate that EBV actively utilizes its miRNAs to flexibly manipulate various viral and cellular functions [24]–[28]. However, these functional studies have only addressed a small fraction of the EBV-encoded miRNAs.

Previously, we utilized next-generation sequencing technology to characterize the EBV miRNA transcriptome in NPC tissues [29] and discovered that three EBV-encoded miRNAs, miR-BART3, miR-BART5 and miR-BART9, were expressed at high levels in all the NPC tissues examined. Recently, miR-BART9 was found to up-regulate LMP1 expression levels and enhance growth rates in NKTCL [30]. However, whether miR-BART9 modulates cellular genes and contributes to NPC pathogenesis is still unknown.

In the present study, we investigated the cellular targets and the biological functions of miR-BART9 in NPC cells. Gain- and loss-of function experiments were performed to characterize the function of miR-BART9 in NPC. In vitro analyses showed that that miR-BART9 enhanced the migration and invasiveness of cultured NPC cells. *In vivo*, we observed that miR-BART9 promoted the metastatic activity of NPC cells in a spontaneous metastasis mouse model. Through bioinformatics analysis and functional verification, we demonstrated that miR-BART9 directly repressed E-cadherin (CDH1), a cell-cell adhesion molecule that is pivotal for maintaining an epithelial cell phenotype and acts as a suppressor of tumor metastasis, to exert its promigratory activity [31]. We further investigated the consequences of miR-BART9-mediated E-cadherin suppression at the molecular and cellular levels and found that miR-BART9 induced a mesenchymal-like phenotype in NPC cells. Our results indicated that miR-BART9 is a prometastatic viral miRNA and provide novel insight into how EBV-encoded miRNAs contribute to NPC pathogenesis.

## Results

### miR-BART9 is highly expressed in NPC tissues and EBV-positive NPC cells

**Figure 1:**
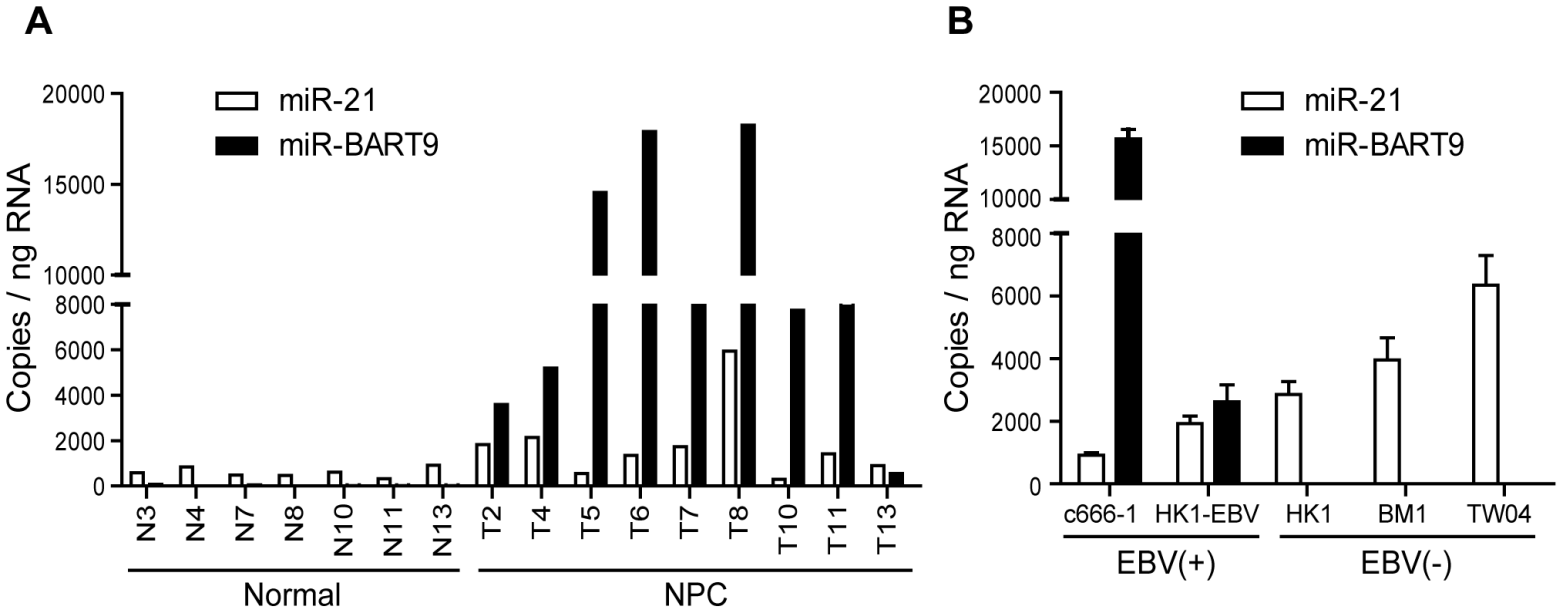
Expression of cellular miR-21 and EBV-miR-BART9 in NPC tissues and cells. (A) Expression levels of miR-21 and miR-BART9 in 9 NPC tumor tissues and 7 adjacent normal tissues. (B) Expression of miR-21 and miR-BART9 in 2 EBV-positive and 3 EBV-negative NPC cell lines. Error bars indicate standard deviations for four replicate assays.

Our deep-sequencing results indicated that miR-BART9 is one of the most abundant EBV miRNAs in NPC tissues. The expression level of miR-BART9 in NPC tissues was higher than that of miR-21, a well-characterized oncogenic human miRNA [29]. To confirm the abundance of miR-BART9 and miR-21 in NPC tissues, we quantified their absolute expression levels via qRT-PCR. Synthetic RNAs corresponding to the mature sequences of miR-BART9 and miR-21were used to establish standard curves for qRT-PCR and the two miRNAs showed a comparable reverse transcription and amplification efficiency in our system (Figure S1). The absolute levels of miR-BART9 and miR-21 were quantified in 7 normal tissues and 9 NPC tissues. As shown in Figure 1A, miR-21 expression was detected in both normal and NPC tissues but was higher in NPC tissues than normal tissues. This result is consistent with previous reports that miR-21 is an oncogenic miRNA whose levels are elevated in NPC tissues [32]. In contrast, miR-BART9 expression was detected in NPC tumors but not in normal tissues. Notably, the expression level of miR-BART9 was higher than that of miR-21 in eight of the nine NPC tissues examined. The obtained data confirmed that miR-BART9 is a highly abundant EBV miRNA in NPC tissues. We also quantified the expression level of miR-BART9 and miR-21 in cultured NPC cells. As shown in Figure 1B, high levels of miR-BART9 were detected in two EBV-positive NPC cell lines (C666-1 and HK1-EBV), which were higher than the observed levels of miR-21. No miR-BART9 signal was detected in EBV-negative NPC cells, including HK1, BM1 and TW04 cells.

### Depleting endogenous miR-BART9 suppresses the migration and invasion of EBV-positive NPC cells

**Figure 2:**
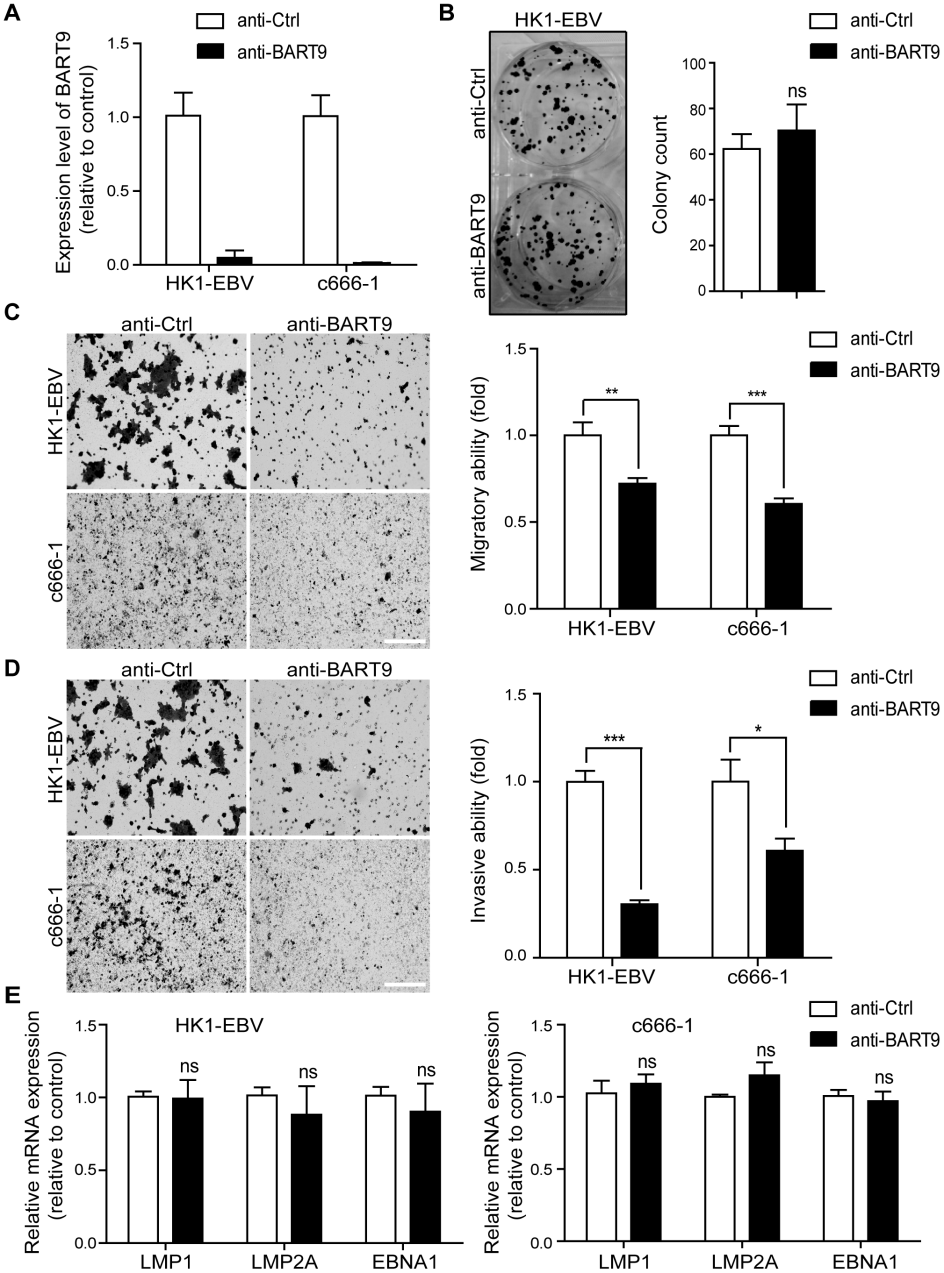
Depletion of endogenous miR-BART9 suppresses the migration and invasiveness of EBV-positive NPC cells. (A) LNA-modified anti-BART9 efficiently decreases the level of mature miR-BART9 in EBV-positive HK1-EBV and C666-1 cells. HK1-EBV and C666-1 cells were treated with a 12.5 nM concentration of an LNA-modified miR-BART9 antisense oligo (anti-BART9) or a scramble control (anti-Ctrl) for 48 hr. The expression level of miR-BART9 was determined via qPCR. (B) HK1-EBV cells were treated with anti-BART9 or anti-Ctrl for 24 hr and the plated for colony formation assays. Colony formation activity was determined via crystal violet staining after 11 days in culture. (C, D) Transwell migration assay (C) and Matrigel invasion assay (D) for HK1-EBV and C666-1 cells. Cells were treated with a 12.5 nM concentration of an LNA-modified miR-BART9 antisense oligo (anti-BART9) or a scramble control (anti-Ctrl) for 48 hr before the migration or invasion assay. Images of cells adhered to the lower surface of the filter insert from a representative experiment are shown. (Left panel). The numbers of migratory or invasive cells were quantified using image J and expressed as the fold change relative to the appropriate cell line (bar graphs). (E) Expression levels of LMP1, LMP2A and EBNA1 in HK1-EBV and C666-1 cells treated with a 12.5 nM concentration of an LNA-modified miR-BART9 antisense oligo (anti-BART9) or a scramble control (anti-Ctrl). Total RNA was collected 48 hr after transfection and mRNA levels were determined via qPCR. The data were normalized to cellular EEF1A1 levels and expressed as the fold change relative to the appropriate cell line. The bar graphs in (B), (C), (D), (E) show means ± SEM from three independent experiments and two-tailed Student's t-tests were performed (*, P<0.05; **, P<0.01; ***, P<0.001).

A previous study by Ramakrishanan et al. found that, in NKTCL cells, miR-BART9 positively regulates the protein and transcript levels of LMP1 and promotes NKTCL cell proliferation [30]. To investigate whether miR-BART9 exerts a similar function in NPC cells, we used an Locked nucleic acid (LNA)-modified anti-BART9 antisense oligo to deplete endogenous miR-BART9 in two EBV-positive NPC cells and analyzed the effect on cell proliferation, migration and invasion. Compared to the scramble control (anti-Ctrl), the anti-miR-BART9 (anit-BART9) oligo effectively reduced mature miR-BART9 levels in C666-1 and HK1-EBV cells by up to 90% (Figure 2A). Importantly, we designed the miR-BART9 sensor to determine the miR-BART9 activity after LNA treatment by luciferase reporter assay. We found that the depletion of miR-BART9 increased luciferase activity in EBV-positive NPC cells by about 3-fold (Figure S2). The results indicated that the suppression of miR-BART9 activities were about 70% with LNA treatment in HK1-EBV and C666-1 cells. The depletion of miR-BART9 had no detectable effect on the growth and proliferation of HK1-EBV cells, as determined in a long-term colony formation assay (Figure 2B). In contrast, the depletion of miR-BART9 significantly reduced the migratory activity of both HK1-EBV and C666-1 cells in Boyden chamber assays (Figure 2C). Similarly, treatment with anti-BART9 suppressed the invasiveness of both HK1-EBV and C666-1 cells in Matrigel-coated Boyden chamber assays (Figure 2D). Previous studies have clearly demonstrated that LMP1, LMP2A and EBNA1 can regulate cell motility in NPC cells and in lymphoblastoid cells [7], [33]. Therefore, we investigated the effect of miR-BART9 depletion on the expression levels of these EBV viral proteins. Quantitative RT-PCR analysis showed that depletion of miR-BART9 did not affect LMP1, LMP2A or EBNA1 transcript levels in either HK1-EBV or C666-1 cells (Figure 2E). In addition, depletion of miR-BART9 did not apparently affect LMP1, LMP2A or EBNA1 protein levels in HK1-EBV cells (Figure S3). These data indicate that miR-BART9 promotes the migration and invasion of NPC cells without affecting cell growth and the promigratory effect of miR-BART9 is independent of LMP1, LMP2A and EBNA1.

### Ectopic expression of miR-BART9 promotes the migration and invasion of EBV-negative NPC cells

**Figure 3:**
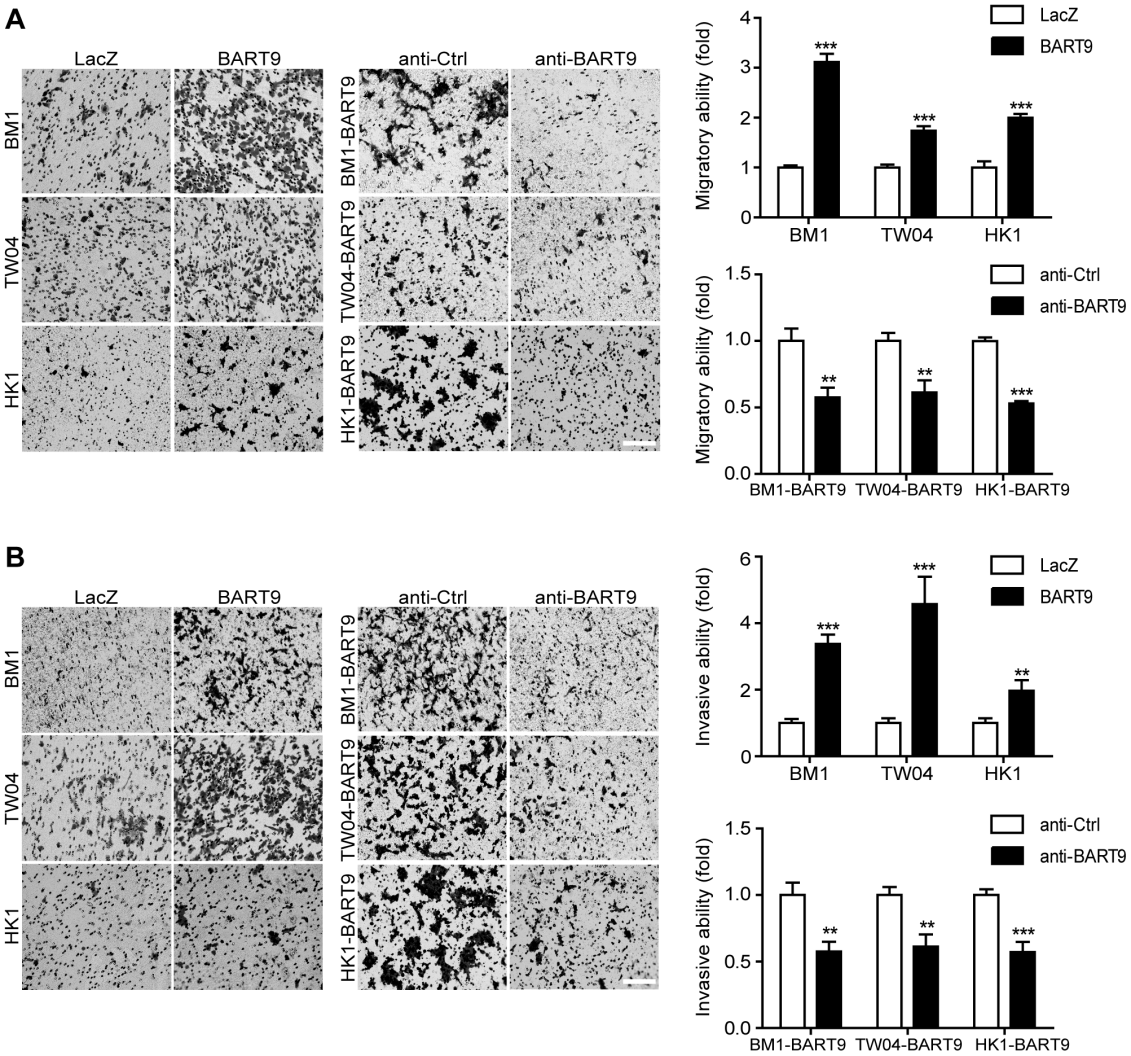
miR-BART9 promotes the migration and invasion of EBV-negative NPC cells. Transwell migration assay (A) and Matrigel invasion assay (B) for miR-BART9- or LacZ-expressing EBV-negative NPC cells. BM1, TW04 and HK1 cells were infected with the miR-BART9 (BART9) or control (LacZ) vector. Cells expressing miR-BART9 or LacZ were directly compared in terms of migratory activity or invasion activity in the same assay (Left panel). In a separate assay, cells expressing miR-BART9 were treated with a 12.5 nM concentration of an LNA-modified miR-BART9 antisense oligo (anti-BART9) or a scramble control (anti-Ctrl) for 48 hr before the migration or invasion assay (middle panel). Images of cells adhered to the lower surface of the filter insert from a representative experiment are shown. The numbers of migratory or invasive cells were quantified using image J and expressed as the fold change relative to the appropriate cell line (bar graphs). The data are expressed as the means ± SEM from three independent experiments and two-tailed Student's t-tests were performed (*, P<0.05; **, P<0.01; ***, P<0.001). Scale bar = 200 µm.

To confirm that miR-BART9 acts as a promigratory miRNA, we established a lentiviral-based system to stably express mature miR-BART9 in three EBV-negative NPC cell lines: HK1, BM1 and TW04. The lentiviral vector contained a GFP-coding region to allow tracking of human NPC cells (Figure S4A). Mature miR-BART9 was readily detectable in all three lentiviral-infected cell lines. The levels of miR-BART9 expression in these three cell lines were comparable to the endogenous level of miR-BART9 in HK1-EBV cells, but lower than that in C666-1 cells (Figures S4B, 1B). The lentiviral-based miR-BART9 expression system was used in all subsequent miR-BART9 gain-of-function analyses. To confirm the miR-BART9 expression, we used the miR-BART9 sensor to determine the luciferase activity after transduction with lenti-miR-BART9 in three NPC cell lines. The exogenous expression of miR-BART9 suppressed the luciferase activities by about 60–70% in lentiviral infected NPC cell lines (Figure S4C). Then we first examined the effect of miR-BART9 on the growth of these EBV-negative NPC cells. Similar to the results of the miR-BART9 depletion study, ectopic expression of miR-BART9 had no significant effect on cell growth and proliferation, as determined via short-term growth curve analysis and long-term colony-forming assays performed in all three types of EBV-negative NPC cells (Figures S5A, S5B). On the other hand, the exogenous expression of miR-BART9 significantly enhanced the migration of cultured BM1, TW04 and HK1 cells in Boyden chamber assays (Figure 3A). The miR-BART9-associated migratory activity observed in these three types of cells was specifically repressed when miR-BART9-expressing cells were treated with anti-BART9, but not with the anti-Ctrl oligo (Figure 3A). The effect of miR-BART9 on cell invasiveness was examined via Matrigel-coated Boyden chamber assays. Similarly, exogenous expression of miR-BART9 was found to significantly increase the invasion capability of the three NPC cell lines (Figure 3B), while miR-BART9-associated invasiveness was specifically suppressed in cells treated with anti-BART9, but not the anti-Ctrl oligo (Figure 3B). To check the knock-down efficiency of miR-BART9, we also used the miR-BART9 sensor to reflect the miR-BART9 activity after LNA treatment in lentiviral-miR-BART9 expressing NPC cells. We found that the depletion of miR-BART9 increased 2–3 folds luciferase activities. The results indicated that the suppression of miR-BART9 activities were about 50–70% with LNA treatment in lentiviral-miR-BART9 expressing NPC cells (Figure S4D). These results clearly demonstrated that ectopic expression of physiological levels of miR-BART9 promotes the migration and invasion capability of EBV-negative NPC cells and confirmed that miR-BART9 is a promigratory viral miRNA.

### Exogenous expression of miR-BART9 promotes the metastasis of EBV-negative NPC cells *in vivo*

**Figure 4:**
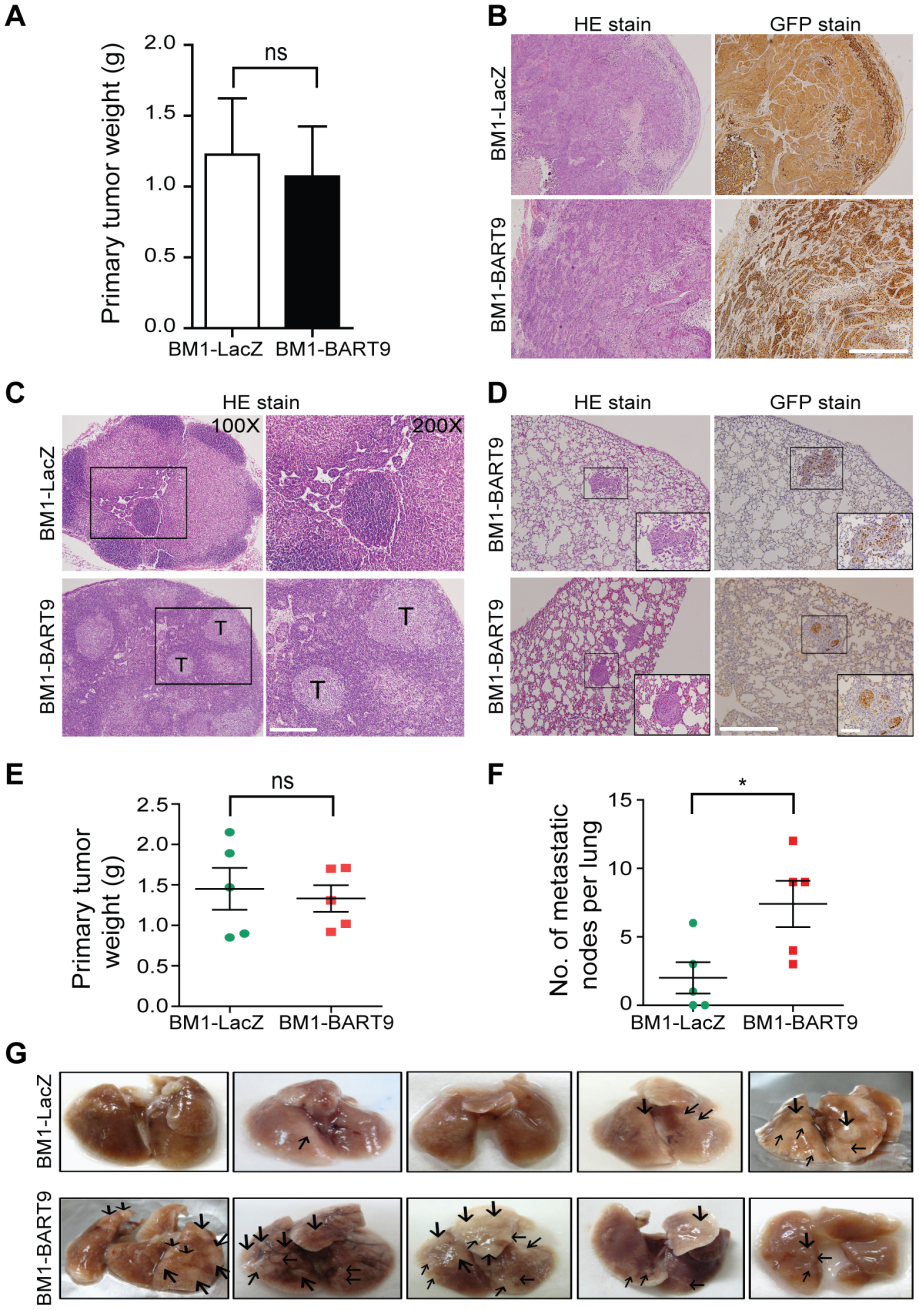
miR-BART9 enhances the metastatic activity of EBV-negative NPC cells *in vivo*. (A) Primary tumor weights in nude mice subcutaneously injected with 1×10^6^ BM1 cells expressing miR-BART9 or LacZ, at 8 weeks after inoculation. The data are presented as the means ± SEM (20 mice per group). (B) Hematoxylin/eosin (HE)-stained (left) and GFP-stained (right) sections of primary tumors isolated from mice that received BM1 cells expressing miR-BART9 or LacZ. Images were acquired at 100×. Scale bar = 500 µm. (C) Representative images of metastatic NPC cells in lymph nodes. HE-stained sections of lymph nodes isolated from mice that received BM1 cells expressing miR-BART9 or LacZ. Rectangular boxes indicate tumor cells in lymph nodes. Images were acquired at 100× and 200×. Scale bar = 200 µm. (D) Representative images of miR-BART9-expressing BM1 cells after metastasis to lung tissue. HE-stained and GFP-stained sections of lung tissues isolated from mice that received miR-BART9-expressing BM1 cells. Rectangular boxes indicate clusters of micrometastatic cells in the lung. Images were acquired at 100× and 400×. Scale bar = 500 µm and 100 µm. (E and F) Weights of primary tumors (E) and numbers of visible lung metastases (F) in nude mice that received 5×10^6^ miR-BART9- or LacZ-expressing BM1 cells injected into the right flank, at week 8 after inoculation. The data are presented as the means ± SEM (each data point represents a different mouse; n = 5 mice per group). P value calculated by two-tailed Student's t-test. (G) Representative images of visible nodules on the surface of the lungs. Arrows indicate clusters of tumor cells that have colonized the lung tissue.

Local invasion and distant metastasis are commonly observed in clinical NPC patients. To determine whether the migration and invasion promoted by miR-BART9 has direct relevance to NPC invasion and metastasis *in vivo*, we inoculated BM1 cells expressing LacZ or miR-BART9 into the right flank of nude mice and investigated their spontaneous metastatic behavior. Mice subcutaneously inoculated with 1×10^6^ BM1-LacZ or BM1-BART9 cells developed palpable tumors after 10 days and their tumors grew at comparable rates up to 8 weeks. At the time of sacrifice, no significant difference in the weight of primary tumor was observed between LacZ- and BART9-expressing BM1 cells (Figure 4A). These results are consistent with the *in vitro* observation that miR-BART9 has no effect on the growth and proliferation of cultured NPC cells (Figures S5A, S5B). Histological examination of the primary tumors using HE and anti-GFP stains revealed that the tumors formed by BM1-BART9 cells were loosely organized, with GFP-negative noncancerous stromal cells being intermixed with GFP-positive tumor cells (Figure 4B). In contrast, the tumors formed by BM1-LacZ cells were compact and showed highly homogenous GFP-staining (Figure 4B).

To evaluate the effect of miR-BART9 on the metastasis of NPC cells, we examined lymph node, lung and liver tissues from individual mice for a GFP signal as a sign of metastasis. Among the 20 mice inoculated with 1×10^6^ BM1-LacZ cells, two showed metastasis to proximal lymph nodes (Figure 4C), while no metastasis to the lungs or liver was detected. In contrast, among the 20 mice inoculated with the same number of BM1-BART9 cells, four mice showed lymph node metastasis (Figure 4C); three mice displayed metastasis to lung (Figure 4D); and one mouse exhibited liver metastasis. Collectively, we found that exogenous expression of miR-BART9 significantly induced spontaneous metastasis of NPC cells *in vivo* (P = 0.03, Chi-square test). To further examine the effect of miR-BART9 on NPC metastasis, we conducted a second experiment by increasing the number of subcutaneously inoculated tumor cells to 5×10^6^ and examined the number of metastasized tumor nodules on the lung surface. Under these conditions, no significant difference in primary tumor weight was observed between BM1-LacZ and BM1-BART9 mice (Figure 4E). Tumor nodules were detected on the lung surface in three of five BM1-LacZ mice, with an average of 2.0±1.1 nodules being recorded per mouse (Figures 4F, 4G). In contrast, lung surface tumor nodules were detected in all five mice inoculated with BM1-BART9 cells, averaging 7.4±1.7 nodules per mice (Figures 4F, 4G). These results confirmed that miR-BART9 promotes the local invasion and distant metastasis of NPC tumors *in vivo* and indicated that miR-BART9 functions as a prometastatic viral miRNA in NPC.

### miR-BART9 directly targets E-cadherin in NPC cells

**Figure 5:**
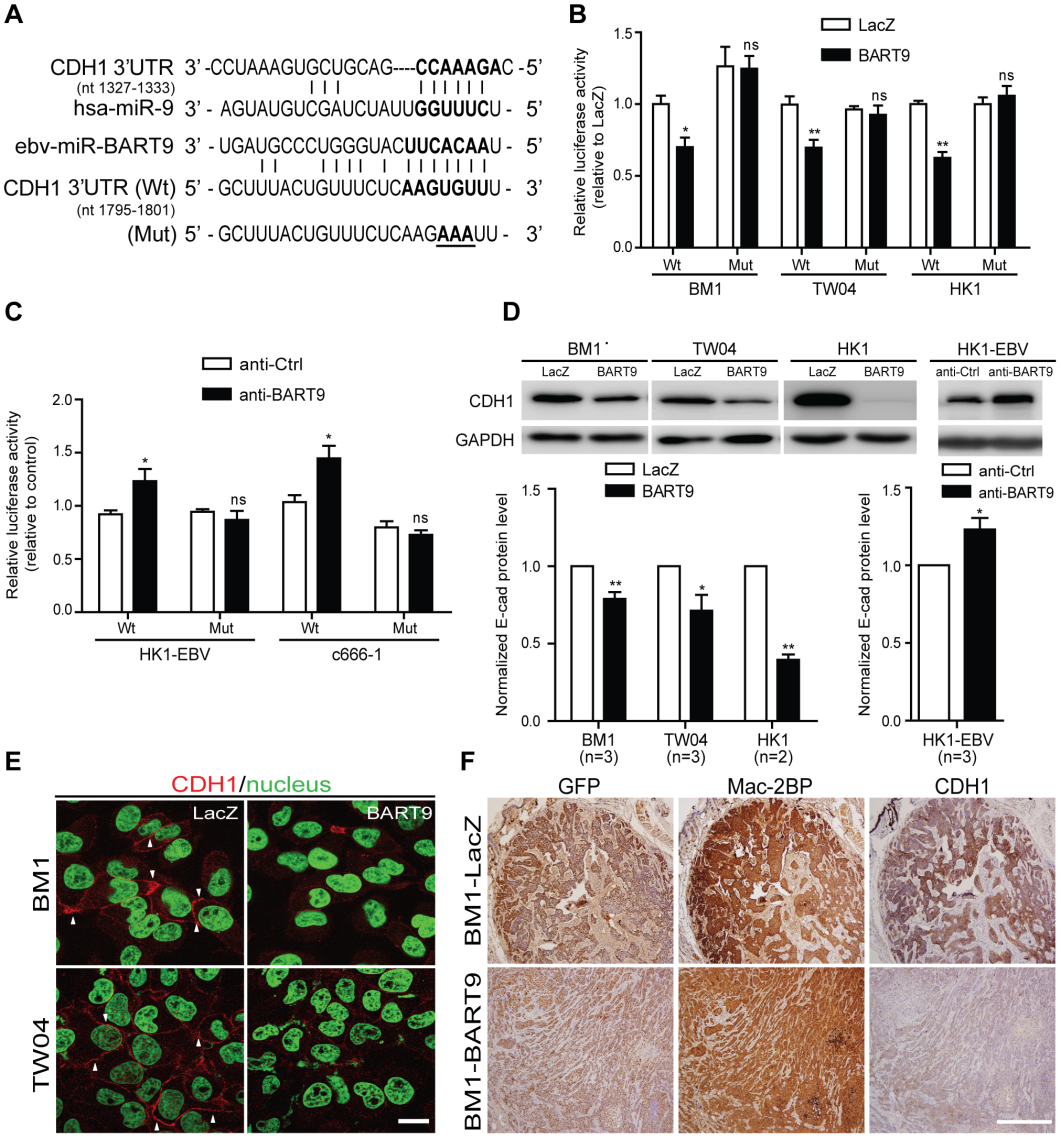
miR-BART9 directly targets E-cadherin. (A) Predicted duplex formation between miR-BART9 and human E-cadherin 3′UTR (Wt). The seed sequence region is highlighted in bold. The putative target sequence of E-cadherin 3′UTR at nt 1795–1801. Mut indicates the mutated E-cadherin 3′UTR sequence used as a control in the reporter assay. Mutated bases are specified by underlining. (B) Luciferase activity of the wild type (Wt) or mutant (Mut) E-cadherin 3′UTR reporter in BM1, TW04 and HK1 cells expressing miR-BART9 or LacZ. (C) Luciferase activity of the wild type (Wt) or mutant (Mut) E-cadherin 3′UTR reporter in HK1-EBV and C666-1 cells treated with a 12.5 nM concentration of an LNA-modified miR-BART9 antisense oligo (anti-BART9) or a scramble control (anti-Ctrl). (D) Top panel: Immunoblotting analysis of E-cadherin in BM1, TW04 and HK1 cells expressing miR-BART9 or LacZ. (Left) or HK1-EBV cells treated with an LNA-modified miR-BART9 antisense oligo (anti-BART9) or scramble control (anti-Ctrl) (Right). GAPDH was used as a loading control. Bottom panel: E-cadherin protein levels were normalized to GAPDH levels, and then compared with the LacZ or anti-Ctrl cells whose normalized levels were expressed as 1.0. Bar graphs provide the means ± SEM of independent experiments and two-tailed Student's t-test were performed (*, P<0.05; **, P<0.01). (E) Representative immunofluorescence staining of E-cadherin and DAPI staining to detect the nucleus in BM1 and TW04 cells expressing miR-BART9 or LacZ. Arrows indicate cell-cell junctions. Scale bar = 20 µm. (F) Representative IHC staining of GFP, human Mac2BP and E-cadherin in sections of primary tumors formed by BM1 cells expressing miR-BART9 or LacZ. Scale bar = 500 µm.

The above functional data indicated that miR-BART9 exerts strong pro-migratory and pro-metastatic activity for NPC. To dissect the mechanisms by which miR-BART9 promotes NPC migration, invasion and metastasis, we next searched potential targets for miR-BART9 in genes involved in NPC pathogenesis. We conducted a computational target prediction for miR-BART9 using the TargetScan algorithm and identified 2173 candidate genes in the human genome as putative miR-BART9 targets. These predicted target genes were subjected to pathway enrichment analysis using KEGG pathway database. Several motility-related pathways, including focal adhesion, ECM-receptor interaction and regulation of actin cytoskeleton, were found to be significantly enriched in the putative targets of miR-BART9 (Table S3). Results from the pathway enrichment analysis was in line with the observed phenotype and implicated that miR-BART9 may directly modulate targets involved in NPC cell motility and metastasis. Previous studies showed that E-cadherin is a key regulator for cell-cell adhesions, cell-extracellular matrix (ECM) interactions and cytoskeleton organization [34]. Down-regulation of E-cadherin is significantly associated with lymph node and distant metastasis in NPC [35]–[37]. Based on the results of target prediction and pathway analysis, we examined whether E-cadherin was involved in the pro-migratory and pro-metastatic effects of miR-BART9. The 3′UTR of E-cadherin contains a single miR-BART9-binding site showing a 7mer-m8 ‘seed match’ and an additional complementary match between nt 12–15 (Figure 5A). To determine whether miR-BART9 directly targets E-cadherin, we performed a 3′UTR reporter assay and found that over-expression of miR-BART9 decreased the activity of a luciferase reporter fused to the wild type E-cadherin 3′UTR, but not to the mutant 3′UTR in the three types of NPC cells (Figure 5B). In contrast, depleting endogenous miR-BART9 in HK1-EBV and C666-1 cells increased the luciferase activity of the wild type construct, but not the mutant E-cadherin 3′UTR construct (Figure 5C). The effect of miR-BART9 on endogenous E-cadherin mRNA and protein levels were examined in multiple NPC cell lines. Ectopic expression of miR-BART9 suppressed E-cadherin mRNA and protein levels in BM1, TW04 and HK1 cells, while treatment with the anti-BART9 oligo increased E-cadherin mRNA and protein levels in HK1-EBV cells (Figures 5D, S6). Based on Figure 5D, we found that exogenous expression of miR-BART9 suppresses endogenous E-cadherin protein levels by 21%, 29% and 60% in BM1, TW04 and HK1 cells, respectively. On the other hand, the depletion of endogenous miR-BART9 increased 23% E-cadherin protein level in HK1-EBV cells. Collectively, the results were consistent with luciferase reporter assay (Figures 5B, 5C) and RT-qPCR analysis (Figure S6). The effect of miR-BART9 on E-cadherin protein levels was further examined via immunofluorescence staining. In both BM1-LacZ and TW04-LacZ cells, an intense E-cadherin signal was detected at cell-cell junctions (Figure 5E). Junction-associated E-cadherin staining was substantially diminished in both BM1-BART9 and TW04-BART9 cells (Figure 5E). These results confirmed that E-cadherin is a target of miR-BART9 and suggest that elevated expression of miR-BART9 may also reduce the level of E-cadherin in NPC tumors. Therefore, we compared E-cadherin levels in tumor xenografts from mice inoculated with BM1-LacZ and BM1-BART9. Serial sections from the same tumor were stained with GFP to confirm the origin of the cells. As shown in Figure 5F, the BM1-BART9 tumor showed a loosely organized tissue architecture, with abundant GFP-negative stromal cells intermixed with GFP-positive NPC cells, while the BM1-LacZ tumor showed a compact structure containing mostly GFP-positive cells, which were clustered together. The staining intensities of GFP and Mac-2BP, a specific marker that is highly expressed in human NPC tissues [38], were similar in both BM1-LacZ and BM1-BART9 tumors (Figure 5F). In contrast, the staining intensity of E-cadherin was greatly reduced in BM1-BART9 tumors (Figure 5F), suggesting that miR-BART9 also reduces E-cadherin levels in NPC tumors *in vivo*.

### E-cadherin plays a critical role in miR-BART9-mediated migration and invasion in NPC cells

**Figure 6:**
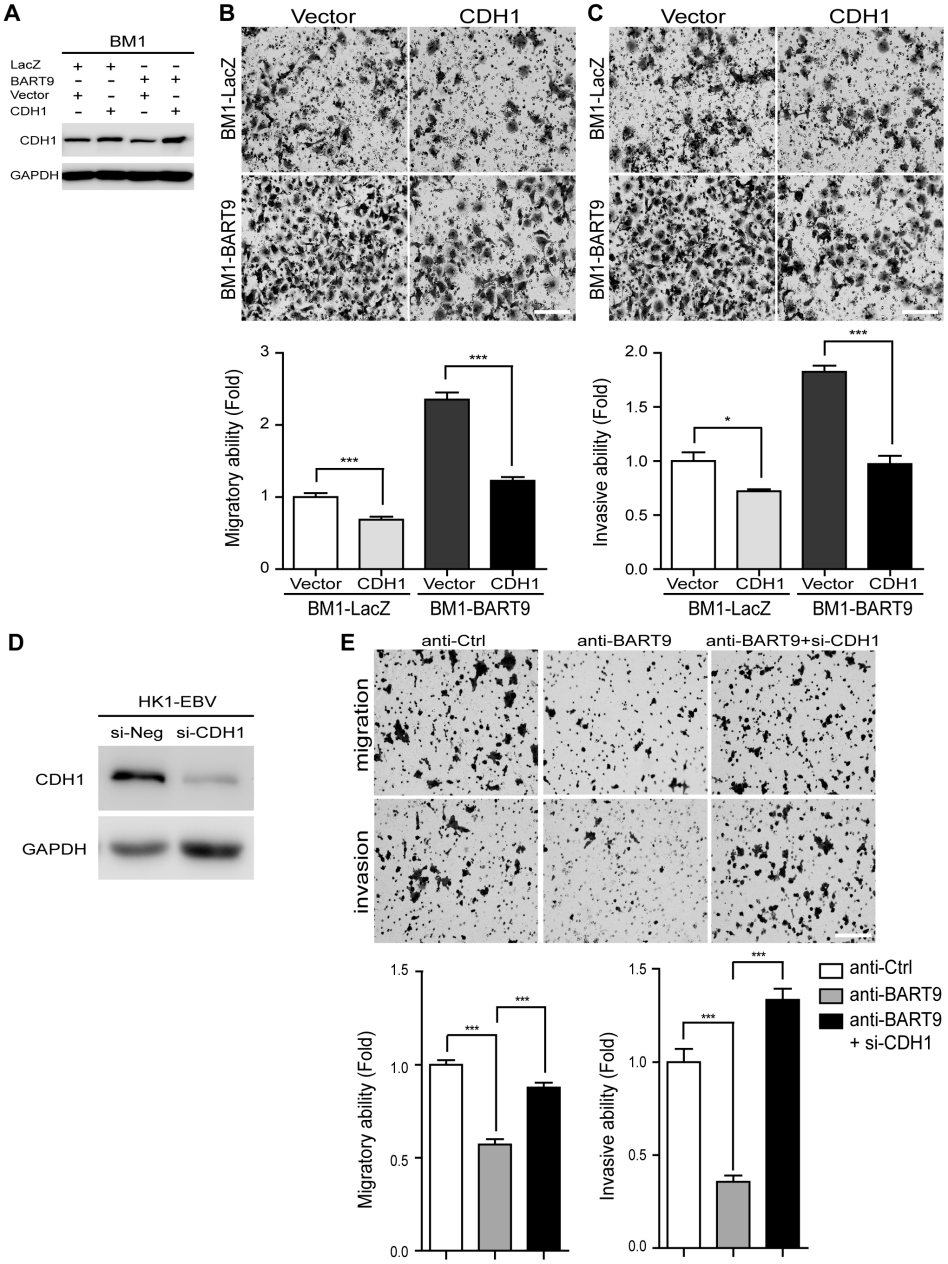
E-cadherin plays a pivotal role in miR-BART9-mediated migration and invasion in NPC cells. (A) Protein level of E-cadherin was increased after introducing pcDNA6/His-CDH1, which contains CDH1 open reading frame without 3′-UTR. Transwell migration assay (B) and Matrigel invasion assay (C) for miR-BART9- or LacZ-expressing BM1 cells with or without ectopic expression of E-cadherin. (D) HK1-EBV cells were transfected with 10 nM siRNA negative control (si-Neg) or CDH1 siRNA (si-CDH1). Expression of E-cadherin was examined by Western blotting. GAPDH was used as a loading control. (E) Transwell migration assay (Upper) and Matrigel invasion assay (Middle) of HK1-EBV cells treated with an LNA-modified miR-BART9 antisense oligo (anti-BART9), scramble control (anti-Ctrl), or anti-BART9 plus E-cadherin siRNA (si-CHD1). Images of cells adhered to the lower surface of the filter insert from a representative experiment are shown. The numbers of migratory or invasive cells were quantified using image J and are expressed as the fold change relative to the appropriate cell line (bar graphs). The data are expressed as the means ± SEM from three independent experiments and two-tailed Student's t-tests were performed (*, P<0.05; ***, P<0.001). Scale bar = 200 µm.

E-cadherin is a membrane protein that mainly localizes to the adhesion junctions of epithelial cells. Previous work has shown that E-cadherin is regulated by a human miRNA, miR-9 (Figure 5A) and down-regulation of E-cadherin promotes cell migration and tumor metastasis in human breast cancer [39]. To determine the role of E-cadherin in miR-BART9-mediated cell migration and invasion, we expressed a plasmid containing only the coding region of E-cadherin in BM1-BART9 and BM1-LacZ cells (Figure 6A) and compared their migration and invasion activities. In BM1-LacZ cells, over-expression of E-cadherin suppressed migratory activity by ∼30% (Figure 6B). Additionally, over-expression of the E-cadherin coding region reduced the migratory capability of BM1-BART9 cells to a level similar to that observed in BM1-LacZ cells (Figure 6B). Similarly, over-expression of E-cadherin suppressed the invasion activity of BM1-LacZ cells and abolished miR-BART9-enhanced invasion in BM1-BART9 cells (Figure 6C). The roles of E-cadherin in miR-BART9-mediated cell migration and invasion were also confirmed in TW04-LacZ and TW04-BART9 cells (Figure S7). These results demonstrated that inhibition of E-cadherin expression is partially responsible for the miR-BART9-mediated biological functions to promote migration and invasion in NPC cells. We further assessed the role of E-cadherin in miR-BART9-enhanced cell migration and invasion in HK1-EBV cells. Depleting endogenous miR-BART9 using the anti-BART9 oligo increased E-cadherin protein levels and reduced the migration and invasion of HK1-EBV cells. The inhibitory effect of the anti-BART9 oligo was blocked by co-treating the cells with si-CDH1 siRNA to deplete E-cadherin in HK1-EBV cells (Figures 6D, 6E). Collectively, these data suggest that E-cadherin plays a pivotal role in miR-BART9-mediated migration and invasion in NPC cells.

### miR-BART9 activates β-catenin and induced a mesenchymal-like morphology

**Figure 7:**
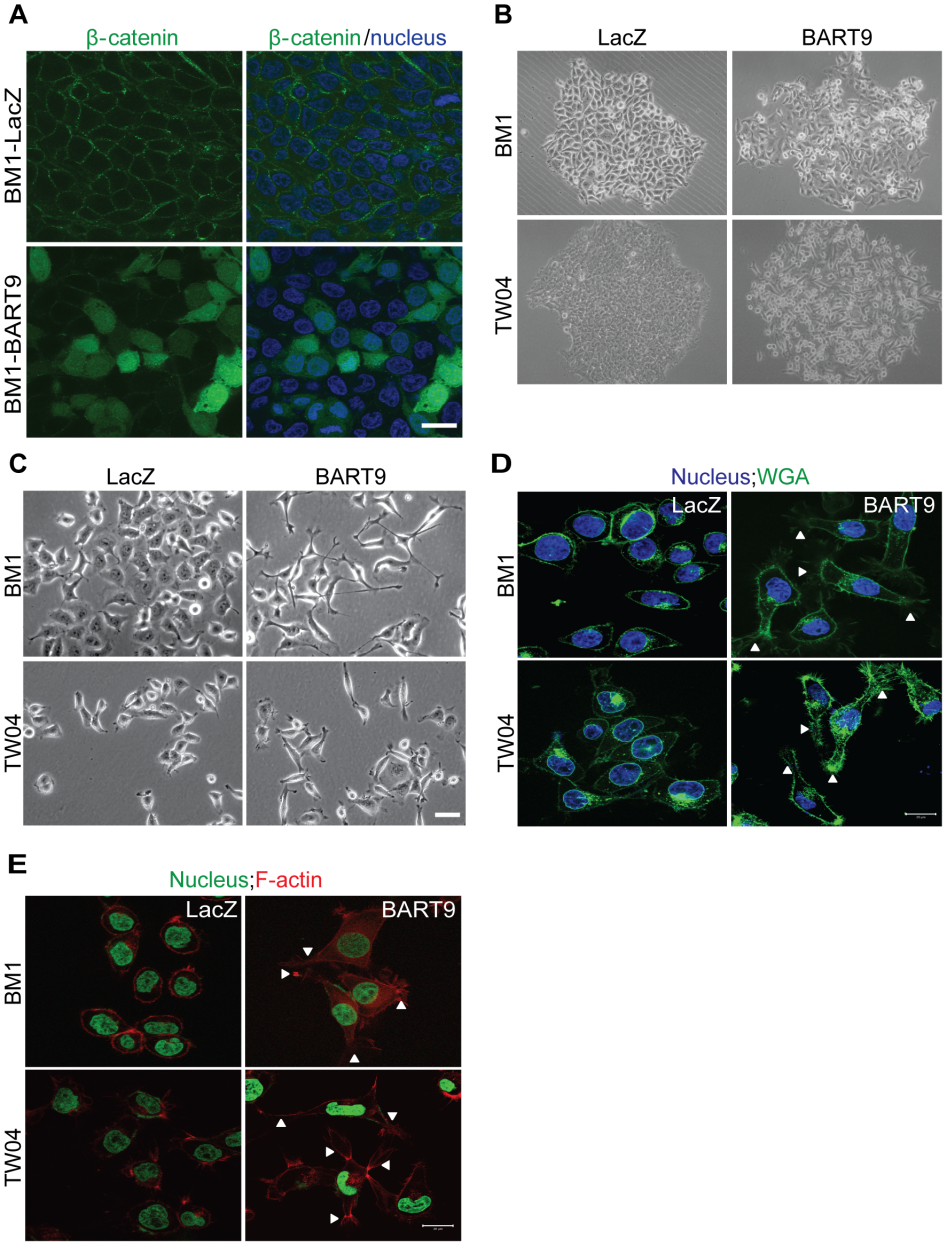
miR-BART9 induces β-catenin translocation and a mesenchymal-like morphology in EBV-negative NPC cells. (A) Representative immunofluorescence staining of β-catenin and DAPI staining to detect the nucleus in BM1 cells expressing miR-BART9 or LacZ. Scale bar = 20 µm. (B) Phase contrast images of BM1 and TW04 cells infected with an miR-BART9-expressing vector (BART9) or control vector (LacZ). Cells were plated in 60-mm dishes at the same density. Images were acquired 9 days after plating. (C) Phase contrast images of BM1 and TW04 cells infected with an miR-BART9-expressing vector (BART9) or control vector (LacZ). Cells were seeded on 60-mm dishes at the same density. Images were acquired 2 days after seeding. Scale bar = 10 µm. (D) DAPI (nucleus) and FITC-conjugated wheat germ agglutinin (WGA) staining in BM1 and TW04 cells expressing the miR-BART9 or control vector (LacZ). Arrowheads indicate filopodia structures. Scale bar = 20 µm. (E) DAPI (nucleus) and Alexa Fluor 594-conjugated phalloidin (F-actin) staining in BM1 and TW04 cells expressing the miR-BART9 or control vector (LacZ). Arrowheads indicate stress fibers. Scale bar = 20 µm.

In normal epithelial cells, E-cadherin is localized at intercellular junctions and is linked to the actin cytoskeleton through β-catenin. Loss of membranous E-cadherin causes a redistribution of β-catenin from the membrane to the cytoplasm and nucleus and is frequently accompanied by a profound morphological change, converting epithelial cells to spindle-shaped fibroblast-like cells with enhanced mobility. To determine whether E-cadherin down-modulation by miR-BART9 leads to β-catenin redistribution, we examined β-catenin localization using immunofluorescence. In confluent BM1-LacZ cells, β-catenin staining was mainly detected in the plasma membrane and clearly outlined cell-cell junctions (Figure 7A). In BM1-BART9 cells, β-catenin staining was mainly detected in the cytoplasm and nucleus, as depicted by the pattern of DAPI co-staining, whereas junctional staining was greatly attenuated (Figure 7A). In fact, well-formed cell-cell junctions were rarely observed in BM1-BART9 and TW04-BART9 cells, even when these cells were maintained in long-term colony culture (Figure 7B). Under these culture conditions, the BM1-LacZ and TW04-LacZ cells displayed a cuboidal shape, which is typically observed in epithelial cells and were closely associated with neighboring cells. In contrast, the BM1-BART9 and TW04-BART9 cells exhibited an elongated, spindle-like morphology, showing minimum contact with neighboring cells (Figure 7B). The differences in the morphology and growth patterns observed in the BM1-LacZ and BM1-BART9 cells under long-term culture appeared to mirror the growth behavior and tissue architecture recorded in the xenograft tumors. To further evaluate the morphological changes associated with miR-BART9 expression, we compared the morphology of BM1-BART9 and TW04-BART9 cells to the corresponding LacZ control cells in low density cultures. Two days after seeding in regular culture plates, the BM1-BART9 and TW04-BART9 cells showed a spindle-like or star-like morphology, with clearly observable filopodia forming in their borders (Figure 7C). Fluorescent wheat germ agglutinin (WGA) staining outlined a spindle-like morphology with protruding filopodia in miR-BART9 cells expressing BM1 and TW04 (Figure 7D). Furthermore, in BM1-BART9 and TW04-BART9 cells, phalloidin staining of F-actin revealed the presence of well-organized actin stress fibers throughout the cells, a feature that is characteristic of migrating mesenchymal cells (Figure 7E). These results indicated that miR-BART9 induced the nuclear translocation of β-catenin and triggered NPC cells to adopt a motile, mesenchymal-like morphology.

### miR-BART9 induces a mesenchymal-like phenotype in NPC cells

**Figure 8:**
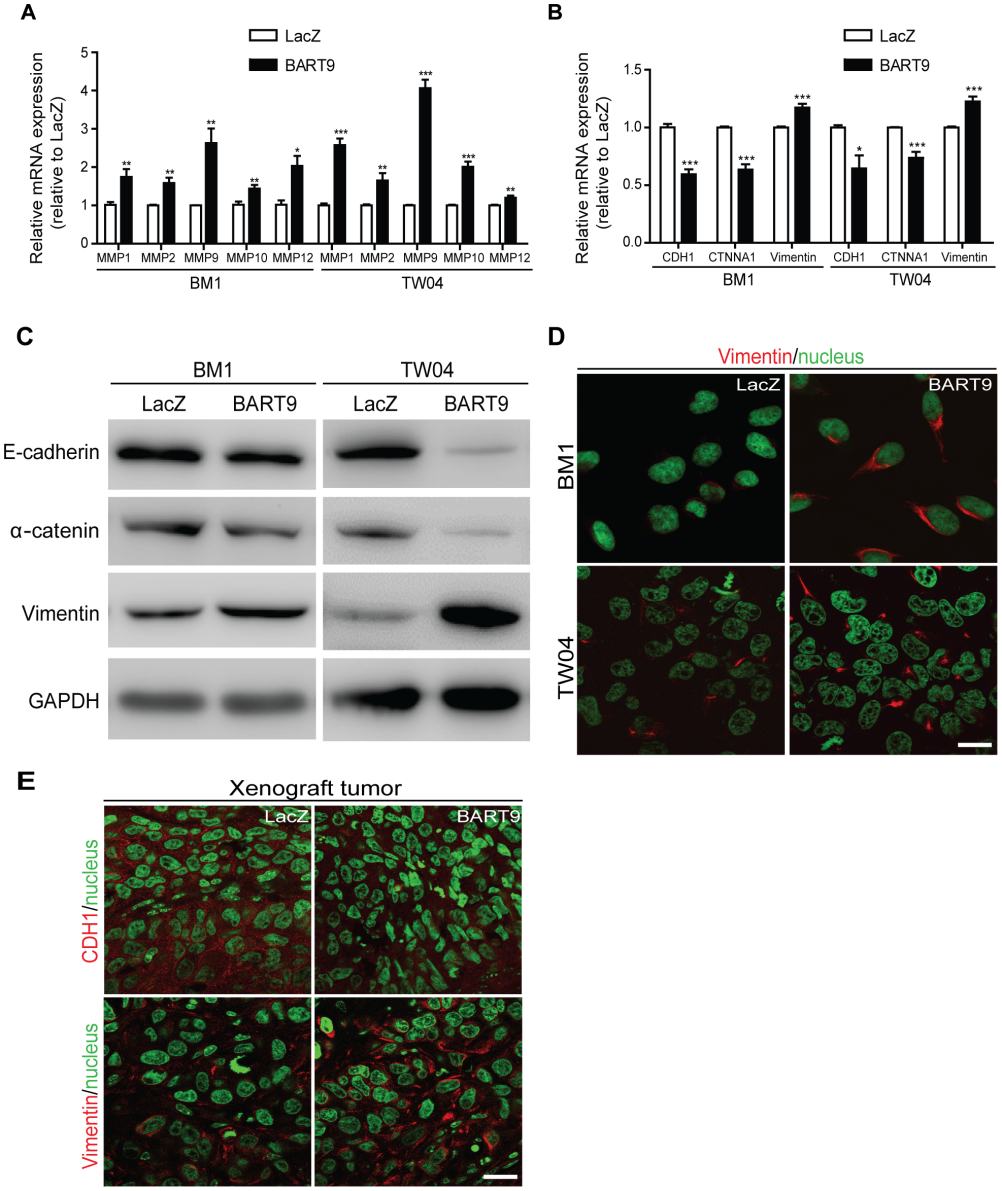
miR-BART9 up-regulates mesenchymal markers in EBV-negative NPC cells. Expression levels of matrix metalloproteases MMP1, MMP2, MMP9, MMP10 and MMP12 (A) and E-cadherin (CDH1), α-catenin (CTNNA1) and vimentin (B) in BM1 and TW04 cells infected with lentivirus expressing the miR-BART9 or control (LacZ) vector. mRNA levels were determined via qPCR. The data were normalized to cellular EEF1A1 levels and expressed as the fold change relative to the appropriate cell line. Bar graphs provide the means ± SEM of three independent experiments and two-tailed Student's t-test were performed (*, P<0.05; **, P<0.01; ***, P<0.001). (C) Western blot analysis for the expression of indicated EMT markers. GAPDH protein was used as a protein loading control. (D) Representative immunofluorescence staining of vimentin and DAPI staining to detect the nucleus in BM1 and TW04 cells expressing miR-BART9 or LacZ. Scale bar = 20 µm. (E) Representative immunofluorescence staining of E-cadherin (CDH1) and vimentin and DAPI staining to detect the nucleus in sections of primary tumors formed by BM1 cells expressing miR-BART9 or LacZ. Scale bar = 20 µm.

E-cadherin plays a central role in maintaining an epithelial phenotype by sequestering β-catenin in the plasma membrane. Loss of E-cadherin and concomitant nuclear translocation of β-catenin have been shown to promote cell migration by disrupting cell-matrix interactions, down-regulating epithelial markers and up-regulating mesenchymal markers. We therefore examined the effect of miR-BART9 on the expression levels of matrix degradation enzymes and common epithelial and mesenchymal markers. Ectopic expression of miR-BART9 in both BM1 and TW04 cells significantly up-regulated the expression levels of multiple matrix metalloproteases, including MMP1, MMP2, MMP9, MMP10 and MMP12, suggesting that miR-BART9 could enhance degradation of the extracellular matrix and promote the motility of NPC cells (Figure 8A). We further analyzed the effect of miR-BART9 on the mRNA and protein levels of α-catenin and vimentin, which are markers of epithelial cells and mesenchymal cells, respectively. Compared to BM1-LacZ cells, BM1-BART9 cells expressed lower levels of α-catenin and higher levels of vimentin, suggesting that miR-BART9 promotes mesenchymal-like characteristics in NPC cells (Figures 8B, 8C). Similar results were observed in TW04-BART9 cells (Figures 8B, 8C). The effect of miR-BART9 on the expression and distribution of E-cadherin and vimentin was further examined via immunofluorescence in cultured BM1-LacZ and BM1-BART9 cells (Figure 8D) and in xenograft tumors (Figure 8E). In both in vitro and *in vivo* models, BM1-BART9 cells showed reduced E-cadherin staining and elevated vimentin staining compared to BM1-LacZ cells. Together, these results indicated that miR-BART9 conferred migratory properties on NPC cells by promoting a mesenchymal-like phenotype.

## Discussion

In a previous sequencing study, we revealed that miR-BART9 is among the most abundantly expressed EBV miRNAs in NPC tumors. In the present study, we conducted TaqMan-based qPCR to determine the absolute level of miR-BART9 and confirmed that in a majority of NPC tissues, the absolute expression level of miR-BART9 was higher than that of a highly abundant cellular oncogenic miRNA, miR-21. To explore the biological function of miR-BART9, we performed loss- and gain-of-function analyses in multiple EBV-positive and EBV-negative NPC cell lines. In EBV-positive NPC cells, depleting endogenous miR-BART9 had no discernable effect on the growth of the cells but significantly reduced their migratory and invasive capability. The observed promigratory and proinvasive effects were confirmed via exogenously expressing miR-BART9 in EBV-negative NPC cells. These miR-BART9-expressing NPC cells also showed a more pronounced metastatic capability in a mouse xenogoraft model. Computational analysis predicted a putative miR-BART9-binding site in the 3′UTR of E-cadherin and a reporter assay confirmed that miR-BART9 directly targeted E-cadherin. In EBV-negative NPC cells, expressing miR-BART9 reduced the expression of E-cadherin and induced a mesenchymal-like phenotype. The observed miR-BART9-enhanced migration and invasion could be reversed by expressing E-cadherin. NPC xenograft tumors expressing miR-BART9 also showed reduced E-cadherin protein levels and an elevated vimentin signal. Together, these data demonstrated that miR-BART9 directly targeted E-cadherin to induce a mesenchymal-like phenotype and promote the migration of NPC cells.

The importance of miR-BART9 in NPC metastasis is underscored by the high level of miR-BART9 expression detected in all the examined NPC tissues. Among head and neck tumors, NPCs are well recognized for their high propensity to undergo nodal and distant metastasis. Previous studies on the metastatic capability of NPC have mainly focused on the roles of two viral oncoproteins: LMP1 and LMP2A. LMP1 has been shown to regulate the expression of multiple metastasis-related genes, including E-cadherin, MMPs, c-Met, VEGF, EGFR and COX-2, by activating the NF-kB, AP-1, MAPK, JAK/STAT and PI3K/AKT signaling pathways [40]. Recently, Liu et al. also demonstrated that LMP1 interacts with FGD4 to activate cdc42 and promote the migration of NPC cells [41]. Although LMP1 is capable of inducing cell growth and transformation, elevated expression of LMP1 can result in growth inhibition and sensitization to apoptosis [42]–[43]. Immunoblotting or immunohistochemical staining analyses have detected the LMP1 protein in 50–70% of NPC cases [5], [44]. The variable LMP1 expression pattern observed in NPC may be due to the down-modulation by several BART miRNAs such as BART5-5p and BART19-5p [17], [27]. Similarly, LMP2A has been shown to induce primary epithelial cell invasion and migration by up-regulating MMPs and αv-integrin through ERK/Fra-1 [45] and ITAM/Sky- and Akt-dependent signaling [46]. LMP2A is a potent immunogenic viral antigen and is recognized by cytotoxic T cells. In NPC tissues, the LMP2A protein is detected in 57.6% of cases [47], presumably due to down-modulation by miR-BART22 to evade the host immune response [18]. Unlike the LMP1 and LMP2A proteins, BART transcripts are consistently detected at high levels in all NPC cases. Our findings indicate that miR-BART9 is a significant viral contributor to the migratory and invasive capability of EBV-infected tumor cells. The promigratory and proinvasive activities of miR-BART9 provide the virus with a non-immunogenic mechanism to regulate the motility of host cells and may explain the highly metastatic behavior of EBV-positive NPC cells observed even in the absence of LMP1 and LMP2A.

In the present study, we showed that over-expressing miR-BART9 significantly reduced the expression of E-cadherin in NPC cells and xenograft tumors, while restoration of E-cadherin completely abolished the promigratory and proinvasive activity of miR-BART9. These data unequivocally confirmed that E-cadherin is a pivotal cellular target of miR-BART9 in NPC cells. E-cadherin is a transmembrane glycoprotein that is predominantly expressed on the surface of epithelial cells. E-cadherin mediates calcium-dependent intercellular adhesion and acts as an invasion/metastasis suppressor gene. In NPC patients, loss of membranous E-cadherin is significantly associated with advanced stages and shorter survival [35]. E-cadherin protein expression has been found to be inversely correlated with lymph node metastasis [48]–[49]. Previous studies have revealed that E-cadherin is down-modulated by EBV oncoproteins in NPC cells through multiple mechanisms. For example, methylation of the CpG islands in the E-cadherin promoter region has been found to be correlated with a loss of E-cadherin mRNA and protein expression in NPC cell lines and tissues [50]–[51]. Tsai et al. later confirmed that this promoter methylation is mediated by LMP1-activated DNA methyltransferase 1 [52]. In addition to promoter methylation, LMP1 also up-regulates snail, a transcription repressor, to repress E-cadherin expression and promote cellular motility and invasiveness in NPC cells [53]. Thus, our observation that miR-BART9 directly down-modulated E-cadherin levels provides a third mechanism exploited by EBV to escape the rigid structural constraints imposed by tissue architecture and to promote the migration of virus-infected epithelial cells. Importantly, LMP1 mainly regulates E-cadherin at the transcriptional level, while miR-BART9 regulates E-cadherin at the post-transcriptional level. Therefore, it is highly probable that these two viral products may function synergistically to repress cellular E-cadherin in EBV-positive NPC cells.

Previously, Ramakrishnan et al. showed that miR-BART9 was highly expressed in cell lines derived from nasal NK/T cell lymphomas, an aggressive type of EBV-associated non-Hodgkin's lymphomas. The authors observed that miR-BART9 positively regulated the mRNA and protein levels of LMP1 and that depleting endogenous miR-BART9 slowed the growth of NKT cells [30]. While the exact mechanism underlying these results is unknown, miR-BART9 appears to be involved in NKTCL proliferation in an LMP1-dependent manner. In the present study, depleting endogenous miR-BART9 did not alter the transcript of LMP1, LMP2A or EBNA1 and had no discernable effect on the growth or proliferation of two EBV-positive NPC cell lines. Then the depletion of miR-BART9 did not apparently affect LMP1, LMP2A or EBNA1 protein levels in HK1-EBV cells (Figure S3). Furthermore, the ectopic expression of miR-BART9 in three EBV-negative NPC cell lines significantly enhanced the migratory activity of all three lines. These results suggest that crosstalk with LMP1 or other EBV oncoproteins is likely not required for miR-BART9 to exert its function in NPC. Although NPC and NKTCL both show a latency II EBV expression pattern and miR-BART9 is highly expressed in both cell types, the roles of miR-BART9 seem to be quite different in these two tumor types, possibly reflecting the differences in cell origin and tumor progression. Whether miR-BART9 regulates any cellular targets or affects NKT migration is currently unknown. On the other hand, Riley et al. indentified that miR-BART9 was one of the 12 most abundant EBV miRNAs in latency III Jijoye BL cells [17]. The authors observed these EBV miRNAs and human miR-17∼92 cluster co-targets human genes that regulate apoptosis, cell cycle, Wnt signaling and transcription pathways in BL cells. However, E-cadherin was not detected in BL cells through High-throughput sequencing and crosslinking immunoprecipitation (HITS-CLIP). The absence of E-cadherin may be due to the differences in cell line characteristic.

In EBV-negative NPC cells, miR-BART9 induced a change in cell shape resulting in cells with a morphology similar to mesenchymal cells. This morphological change as accompanied by a reduction of membrane E-cadherin levels and rearrangement of actin filaments. The observed cytoskeletal reorganization, together with the translocation of beta-catenin from the cytoplasm to the nucleus, up-regulation of MMPs and acquisition of the mesenchymal marker vimentin, indicated that miR-BART9 induced a mesenchymal-like phenotype in cultured NPC cells. Previous studies have suggested that the acquisition of a mesenchymal phenotype is an important mechanism whereby carcinomas progress to a metastatic stage. Loss of e-cadherin and nuclear translocation of β-catenin have been shown to be correlated with the acquisition of an invasive phenotype in several types of cancers, including NPC [35]–[37]. Our data indicated that miR-BART9 may facilitate the metastatic behavior of NPC cells by directly down-regulating E-cadherin and inducing a mesenchymal-like phenotype to generate motile cells. Both LMP1 and LMP2A have been shown to induce a mesenchymal-like phenotype in cultured NPC cells. Our data indicated that, in addition to viral oncoproteins, miR-BART9 functions as a prometastatic miRNA by promoting a mesenchymal-like phenotype in NPC cells.

Alteration of epithelial-mesenchymal plasticity has been recognized as a central mechanism through which epithelial carcinomas generate motile cells that can migrate from the primary tumor to a new tissue site. Several cellular miRNAs have been implicated in the regulation of epithelial-to-mesenchymal transition. In particular, the miR-200 family has been shown to prevent epithelial-mesenchymal transition by targeting the E-cadherin transcriptional repressors ZEB1 and ZEB2. Thus, the miR-200 family is regarded as a gatekeeper of the epithelial phenotype. Down-regulation of miR-200 family members promotes mesenchymal characteristics in cancer cells and has been associated with a metastatic phenotype in various cancers [54]–[55]. In previous studies, we have shown that the expression level of miR-200 is significantly down-modulated in NPC tumors [29], [32]. In the present study, we demonstrated that the EBV-encoded miRNA miR-BART9 was highly expressed in NPC tissues and that miR-BART9 directly targeted E-cadherin to promote a mesenchymal phenotype and migratory activity in NPC cells. The high abundance of viral miR-BART9, coupled with a down-modulation of cellular levels of miR-200 family members in EBV-positive NPC tumors, could drive epithelial carcinoma cells to adopt a mesenchymal phenotype with greatly enhanced motility and contribute to the highly metastatic behavior of NPC tumors. It is worth noting that miR-BART9 and the miR-200 family share the same “seed sequence”, which is a critical region for the interaction between an miRNA and its target. Whether the viral miRNA miR-BART9 mimics or competes with cellular miRNA miR-200 family members to exert its influence on critical cellular characteristics, such as epithelial or mesenchymal phenotypes, remains to be determined.

## Materials and Methods

### Ethics statement

This research followed the tenets of the Declaration of Helsinki and all subjects signed an informed consent form approved by the Institutional Review Board of Chang Gung Memorial Hospital before their participation in this study as well as for the use of tissue samples collected prior to treatment. The study protocol was approved by the Medical Ethics and Human Clinical Trial Committee of the Chang Gung Memorial Hospital (IRB No. 98-2729B). All animal experiments were approved and performed according to the United States National Institutes of Health guidelines and the Chang Gang Institutional Animal Care and Use Committee Guide for Care and Use of Laboratory animals (IACUC Approval No. CGU11-005).

### Clinical samples

Human nasopharyngeal carcinoma and adjacent non-tumor nasopharynx tissues were obtained from patients undergoing surgery at Chang Gung Memorial Hospital as described previously [29], [32]. The study protocol was approved by the Medical Ethics and Human Clinical Trial Committee of the Chang Gung Memorial Hospital (IRB No. 98-2729B).

### Cell lines

The EBV-positive NPC cell lines C666-1 and HK1-EBV and the EBV-negative NPC cell line HK1 were generous gifts from Dr. Sai-Wah Tsao (University of Hong Kong, China). The NPC-BM1 epithelial cell line was established from a bone marrow biopsy from a female Taiwanese patient with NPC [56]. These cell lines were maintained in RPMI-1640 medium supplemented with 10% fetal calf serum, 2 mM sodium pyruvate and 2 mM L-glutamate. The NPC-TW04 cell line was established using NPC biopsy specimens collected from NPC patients and was maintained in Dulbecco's Modified Eagle's Medium (DMEM) supplemented with 10% fetal bovine serum (FBS) at 37°C in a 5% CO_2_ environment [57]. Unless otherwise specified, all reagents were purchased from Invitrogen (Carlsbad, CA, USA).

### Preparation of total RNA

Total RNA, including both mRNAs and microRNAs, was extracted from clinical tissues and cultured cells using the TRIzol reagent (Invitrogen, Carlsbad, CA, USA) according to the manufacturer's protocol. The concentration of purified total RNA was determined using a NanoDrop Spectrophotometer (Thermo Scientific). RNA integrity was evaluated using an Agilent 2100 BioAnalyzer (Agilent Technologies, Palo Alto, CA, USA). Aliquots of total RNA were used directly for mRNA or microRNA quantification.

### Quantitative RT-PCR

For microRNA quantification, 1 µg of total RNA was converted into cDNA using microRNA-specific stem-looped RT primers and Superscript III reverse transcriptase (Invitrogen, Carlsbad, CA) as described previously [29]. MicroRNA expression levels were quantified using TaqMan probes under the following PCR conditions: 95°C for 10 min, followed by 40 cycles of 94°C for 15 sec and 62°C for 30 sec and a dissociation stage. Synthetic miRNAs were reverse transcribed and amplified under the same conditions to generate standard curves for the calculation of copy numbers. All of the miRNA-specific primers and synthetic miRNAs used in this study are listed in the Table S1.

For mRNA quantification, 1 µg of total RNA was employed as a template for reverse transcription using an oligo dT primer and Superscript III (Invitrogen). Quantitative real-time PCR analysis was performed using SYBR green master mix (ABI) under the following conditions: 95°C for 10 min, followed by 45 cycles of 95°C for 15 sec and 60°C for 1 min. A melting curve analysis was conducted to determine the specificity of the obtained products. The primer sequences used in the qRT-PCR experiments are presented in Table S2. The levels of individual target mRNAs were normalized to those of the endogenous control mRNA EEF1A1 in each sample. All qRT-PCR amplifications were performed using the ABI Prism 7500 Fast Real-Time PCR system (Foster City, CA, USA).

### Establishment of a lentiviral-based miR-BART9 expression system

A double-stranded oligonucleotide with a sequence corresponding to the miR-BART9 precursor was synthesized and inserted into the pcDNA6.2-GW/EmGFP-miR vector under control of the CMV promoter. The cloning primers used here are listed in Table S1. Following confirmation via sequencing, the miR-BART9 expression vector was transferred to a lentiviral expression plasmid (pLenti6/V5-DEST) using Gateway recombination technology (Invitrogen) according to the manufacturer's instructions. Then, lentivirus was produced in HEK293-FT packaging cells by co-transfecting the miR-BART9 expression plasmid or control (LacZ) plasmid with packaging vectors (ViraPower packaging mix, Invitrogen) using Lipofectamine 2000 (Invitrogen). Lentivirus was harvested from clarified culture supernatants 48 hr after transfection and NPC cells were transduced overnight in the presence of 4 µg/mL of polybrene.

### Transient transfection

All plasmid transfections were carried out using Lipofectamine 2000 (Invitrogen), according to the manufacturer's instructions. To conduct a CDH1 rescue experiment, cells were transfected with 10 ng pcDNA6/His-CDH1 (no 3′-UTR) or with the pcDNA6/His vector as a control (a gift from Dr. Sheng-Chieh Hsu, Chung Gung University). All synthetic oligonucleotides and siRNA transfections were conducted using the RNAiMAX reagent (Invitrogen) according to the manufacturer's instructions. For miRNA knockdown analysis, a locked nucleic acid (LNA)-modified miR-BART9 antisense oligo or scrambled control oligo was heated at 95°C for 1 min and then chilled on ice for 3 min prior to use and 12.5 nM was the final concentration of the LNA-modified oligos. To deplete endogenous E-cadherin, si-CDH1 or si-Ctrl was used at a final concentration of 10 nM. All biochemical and biological analyses were performed 48 hr after transfection.

### Transwell migration assay and invasion assay

The migration assay was performed using 24-well transwell inserts with an 8 µm pore size (Corning, Corning NY). The invasion assay was performed by precoating the transwell inserts with Matrigel Basement Membrane Matrix (BD Biosciences, San Diego, CA, USA), according to the manufacturer's instructions. Cells suspended in serum-free medium were seeded in the upper chamber and allowed to transmigrate towards the bottom chamber, containing medium with 10% FBS, for 24 hr (BM1, TW04, HK1, HK1-EBV) or 48 hr (C666-1). The membrane inserts were then fixed and stained with 1% crystal violet. Cells adhered to the lower surface of membrane inserts were visualized via microscopy and the average number of migrated cells was calculated from twelve representative fields on triplicate inserts using ImageJ software.

### Cell proliferation assay and colony formation assay

For growth curve analysis, cells were seeded in sextuplicate at a density of 1×10^3^ cells per well in 96-well plates. The cells were then fixed on days 1, 2 and 4 with 3.7% formaldehyde and stained with 4′, 6-diamidino-2-phenylindole (DAPI). The cell numbers in each well were quantified using the INCell Analyzer 1000 cellular imaging system (GE Healthcare Life Sciences, Buckinghamshire, UK). For the colony formation assay, cells were seeded in triplicate at a density of 200 (HK1-EBV) or 100 cells (BM1, TW04, HK1) per well in six-well plates and cultured with complete medium for 10 to 14 days. The cells were then washed, fixed and stained with 1% crystal violet for 30 minutes. After washing out the dye, the plates were photographed and the number of visible colonies was quantified using the BioSpectrum Imaging System.

### Tumor formation, necropsy and histopathology

Nude mice were purchased from the National Laboratory Animal Center and maintained in microisolator cages. Tumor cells (1×10^6^ or 5×10^6^ cells) were suspended in 100 µl of PBS and inoculated subcutaneously into the right flank of 4-week-old nude mice. All mice were sacrificed 8 weeks after inoculation. The primary tumor, lymph node, lung and liver tissues of each mouse were removed, weighed, photographed and embedded in 10% paraffin. For mice injected with 1×10^6^ tumor cells, each section from the lymph node, lung and liver tissues was subjected to H&E staining for histological examination and evaluation of metastasis. Sections of primary tumors and lung tissues were used to detect the expression of markers (GFP, Mac2BP, E-cadherin) via IHC. For mice injected with 5×10^6^ tumor cells, the primary tumors and the lung tissues were removed, weighed and photographed. The numbers of visible lung-surface metastases in each mouse were recorded.

### Target prediction and pathway enrichment analysis

To perform target prediction, the 3′-UTR sequence of 17330 human protein-coding genes was retrieved from the UCSC genome database, and the sequences of mature miR-BART9 was downloaded from miRBase (http://www.mirbase.org/). Target prediction and context score analyses were performed using the TargetScan_50.pl and TargetScan_Context_Scores.pl scripts downloaded from the TargetScan website (http://www.targetscan.org/vert_50/). A sum context score <−0.15 (non species conserved) was used as a filter to select high efficacy targets. Collectively, a total of 2173 predicted miR-BART9 targets were retrieved. These predicted target genes were subjected to pathway enrichment analysis using DAVID Bioinformatic Resources (http://david.abcc.ncifcrf.gov/, version 6.7) and KEGG pathway database (Table S3).

### Dual luciferase reporter assay

A luciferase reporter construct for the wild type CDH1 3′-UTR containing the predicted binding site for miR-BART9 was cloned into the pMIR-REPORT vector (Ambion) and a mutant CDH1 3′-UTR plasmid was generated by changing the sequence from AAGUGUU to AAGAAAU using the QuickchangeXL Mutagenesis Kit. NPC cells were transfected with a luciferase reporter vector containing either the wild type or mutant E-cadherin 3′UTR using Lipofectamine 2000 (Invitrogen). Cell lysates were harvested 24 hr after transfection and luciferase activity was measured by using the Dual-Luciferase Reporter Assay system (Promega). *Renilla* luciferase activity was used to normalize the firefly luciferase activity values.

### Western blotting

Cell lysates were prepared, quantified and immobilized on PDVF membranes as described previously. The membranes were incubated with a rabbit monoclonal anti-CDH1 (E-cadherin) antibody (1∶5000; Cell signaling #4065), anti-LMP1 monoclonal antibody (S12) (1∶1000; a gift from Dr. Yu-Sun Chang, Chang Gung University), rat monoclonal anti-LMP2A antibody (1∶1000; Bio-Rad MCA2466), anti-EBNA1 antibody (1∶1000; a gift from Dr. Mei-Ru Chen, National Taiwan University) followed by horseradish peroxidase-conjugated goat anti-rabbit IgG or goat anti-mouse IgG (1∶10000; GeneTex #213110-01, #213111-01) and the resultant bands were detected using ECL reagents (Millipore). An anti-glyceraldehyde-3-phosphate dehydrogenase (GAPDH) antibody (1∶10000; BioWorld) was employed as a loading control. Western blot images were captured with the UVP BioSpectrum 600 Imaging System (Upland, CA, USA), and the band intensity was quantified with the Image J software.

### Immunofluorescence

Cells grown on coverslips were fixed in 4% paraformaldehyde, permeabilized with 0.2% Triton X-100, blocked with 0.5% BSA/PBS and incubated with the appropriate primary antibody at 4°C overnight. The primary antibodies included CDH1 (E-cadherin, Cell Signaling #4065, 1∶100), β-catenin (Santa Cruz SC-7963, 1∶100) and vimentin (Cell Signaling #5741, 1∶100) antibodies. After washing, the cells were incubated with an Alexa Fluor 594- or 488-lalebed secondary antibody (Invitrogen) for 1 hr, counterstained with DAPI and mounted with SlowFade Gold antifade reagent (Invitrogen). The images were visualized via confocal microscopy using a ZEISS LSM510 META laser-scanning confocal microscope (Carl Zeiss, Germany) with a 63×1.32 NA oil-immersion objective. For WGA staining, cells were incubated with Alexa Fluor555-labeled WGA (Invitrogen, 1∶800) for 10 min at 37°C prior to washing and fixation. For F-actin staining, cells were fixed, permeabilized and incubated with Alexa594-phalloidin (Invitrogen, 1∶1000) for 20 min at 4°C. After washing, the cells were counterstained with DAPI and mounted.

### Immunohistochemistry

Slide-mounted tissue sections were treated with proteinase K at room temperature for 15 min and incubated with 3% H_2_O_2_ (DAKO) to inhibit endogenous peroxidase activity. Nonspecific binding was blocked with Antibody Diluent and Background Reducing Component (DAKO). The sections were then incubated with an anti-CDH1 (E-cadherin, Cell signaling #4065; 1∶50 dilution), anti-GFP (GeneTex, 1∶50 dilution) or anti-Mac-2BP antibody (a gift from Dr. Jau-Song Yu, Chung Gung University) at room temperature for 1 hr. After washing, the slides were incubated with an HRP-conjugated secondary antibody at room temperature for 20 min and developed with 3,39-diaminobenzidine tetrahydrochloride (DAB) as a chromogen. All images were acquired on an Olympus BX51 microscope (Olympus, Japan).

### Statistical analysis

Statistical analyses were performed with a two-tailed Student's t-test for independent samples, assuming equal variances in all data sets. P<0.05 was considered statistically significant.

### Accession numbers

The Entrez Gene ID numbers for genes or proteins mentioned in the text are 3783750 (LMP1), 3783751 (LMP2A), 3783774 (EBNA1), 999 (E-cadherin), 7431(Vimentin), 1495 (CTNNA1), 2957 (GAPDH).

## Supporting Information

Figure S1 **Standard curves for miR-BART9 and miR-21 qPCR assays.** cDNA samples containing known copy numbers of synthetic miRNAs (3×10^5^ to 3×10^9^ copies) were used to test the absolute sensitivity of each qPCR assay. Standard curves obtained for miR-BART9 and miR-21 by plotting the cycle threshold (CT) values against log input RNA copy number are shown.
(TIF)

Figure S2 **Determine the miR-BART9 activity after LNA treatment in EBV-positive NPC cells.** To verify the knock-down efficiency of miR-BART9 in EBV-positive NPC cells, the miR-BART9 sensor luciferase reporter plasmid was used. Two copies of anti-sense miR-BART9 sequences which perfectly complementary to miR-BART9 were cloned into the pMIR-REPORT vector. HK1-EBV and C666-1 cells were co-transfected with a miR-BART9 sensor luciferase reporter vector and a 12.5 nM concentration of an LNA-modified miR-BART9 antisense oligo (anti-BART9) or a scramble control (anti-Ctrl) using Lipofectamine 2000 (Invitrogen). Cell lysates were harvested 48 hr after transfection and luciferase activity was measured by using the Dual-Luciferase Reporter Assay system (Promega). Luciferase activity was normalized to that of *Renilla* and calculated as a relative fold to control cells (anti-Ctrl). All data are presented as means values ± SEM of three independent experiments. *, *P*<0.05; ***, *P*<0.001.
(TIF)

Figure S3 **Depletion of miR-BART9 did not apparently affect LMP1, LMP2A or EBNA1 protein levels in HK1-EBV cells.** HK1-EBV cells were treated with a 12.5 nM concentration of an LNA-modified miR-BART9 antisense oligo (anti-BART9) or a scramble control (anti-Ctrl) using Lipofectamine 2000 (Invitrogen). After 48 hours, cell lysates were harvested and performed western blots to determine the expression levels of LMP1, LMP2A and EBNA1. GAPDH protein was used as a protein loading control. LMP1, LMP2A and EBNA1 protein levels were normalized to GAPDH levels, and then compared with the anti-Ctrl cells whose normalized levels were expressed as 1.0.
(TIF)

Figure S4 **Expression levels of miR-BART9 in 3 EBV-negative NPC cell lines infected with lentivirus expressing the miR-BART9 or control (miR-LacZ) vector.** (A) HK1, BM1 and TW04 NPC cells were transduced with lentivirus- expressing miR-BART9 vector, then photographed under an inverted fluorescence microscope (×100). The infection efficiency of lentivirus was over 90%. Importantly, no significant cell death was observed after virus infection. (B) After transduction with miR-BART9 LV and miR-LacZ in HK1, BM1 and TW04 NPC cells, miR-BART9 expression levels were detected by real-time RT-PCR analysis in the three cell lines. Error bars indicate standard deviations for four replicate assays. (C) Determine the miR-BART9 activity after infection with lentivirus expressing the miR-BART9 or control (miR-LacZ) vector in three EBV-negative NPC cell lines by luciferase reporter assay. (D) Determine the miR-BART9 activity after treating with a 12.5 nM concentration of an LNA-modified miR-BART9 antisense oligo (anti-BART9) or a scramble control (anti-Ctrl) in three miR-BART9 expressing NPC cells. Luciferase activity was normalized to that of *Renilla* and calculated as a relative fold to LacZ or control cells (anti-Ctrl). All data are presented as means values ± SEM of three independent experiments. **, *P*<0.01; ***, *P*<0.001.
(TIF)

Figure S5 **miR-BART9 has no significant effect on NPC cell growth** ***in vitro*** **.** (A) BM1, TW04 and HK1 cells infected with lentivirus containing the miR-BART9 or control (LacZ) vector and miR-BART9 were plated in 96-well plates. Cells were fixed and stained with DAPI on days 1, 2 and 4. Cell numbers were determined using INCell 1000. The data were expressed as the mean cell counts ± SEM from six wells and a two-tailed Student's t-test was performed. (B) BM1, TW04 and HK1 cells infected with lentivirus containing the miR-BART9 or control (LacZ) vector and miR-BART9 were plated in 6-well plates. Colony formation activity was determined via crystal violet staining after 10 days in culture. The data are expressed as the mean colony counts ± SEM from three independent experiments and a two-tailed Student's t-test was performed.
(TIF)

Figure S6 **E-cadherin mRNA levels were decreased and increased in miR-BART9-overexpressing and knockdown NPC cells.** Left panel: E-cadherin mRNA expression was measured with qRT-PCR in miR-BART9-overexpressing NPC cells. Right panel: Determine the mRNA level of E-cadherin in miR-BART9-depleting HK1-EBV cells.
(TIF)

Figure S7 **Rescue of E-cadherin reversed the effects of miR-BART9 on migration and invasion in NPC TW04 cells.** (A) Rescue of E-cadherin expression in miR-BART9-expressing TW04 cells. Transwell migration assay (B) and Matrigel invasion assay (C) for miR-BART9- or LacZ-expressing TW04 cells, with or without ectopic expression of E-cadherin. Images of cells adhered to the lower surface of the filter insert from a representative experiment are shown. The numbers of migratory or invasive cells were quantified using image J and expressed as the fold change relative to the appropriate cell line (bar graphs). The data are expressed as the means ± SEM from three independent experiments and two-tailed Student's t-tests were performed (*, P<0.05; **, P<0.01; ***, P<0.001). Scale bar = 200 µm.
(TIF)

Table S1 **Synthetic RNA; LNA; Cloning primers.**
(PDF)

Table S2 **Primers for qPCR analysis.**
(PDF)

Table S3 **Enrichment analysis of predicted miR-BART9 targets in KEGG cell signaling pathway database.**
(PDF)
