# Supplementary figures and images for: The Epstein-Barr Virus-Encoded MicroRNA MiR-BART9 Promotes Tumor Metastasis by Targeting E-Cadherin in Nasopharyngeal Carcinoma
Source: PLoS Pathog. 2014 Feb 27;10(2):e1003974. doi: 10.1371/journal.ppat.1003974 (PMC3937311; doi:10.1371/journal.ppat.1003974)

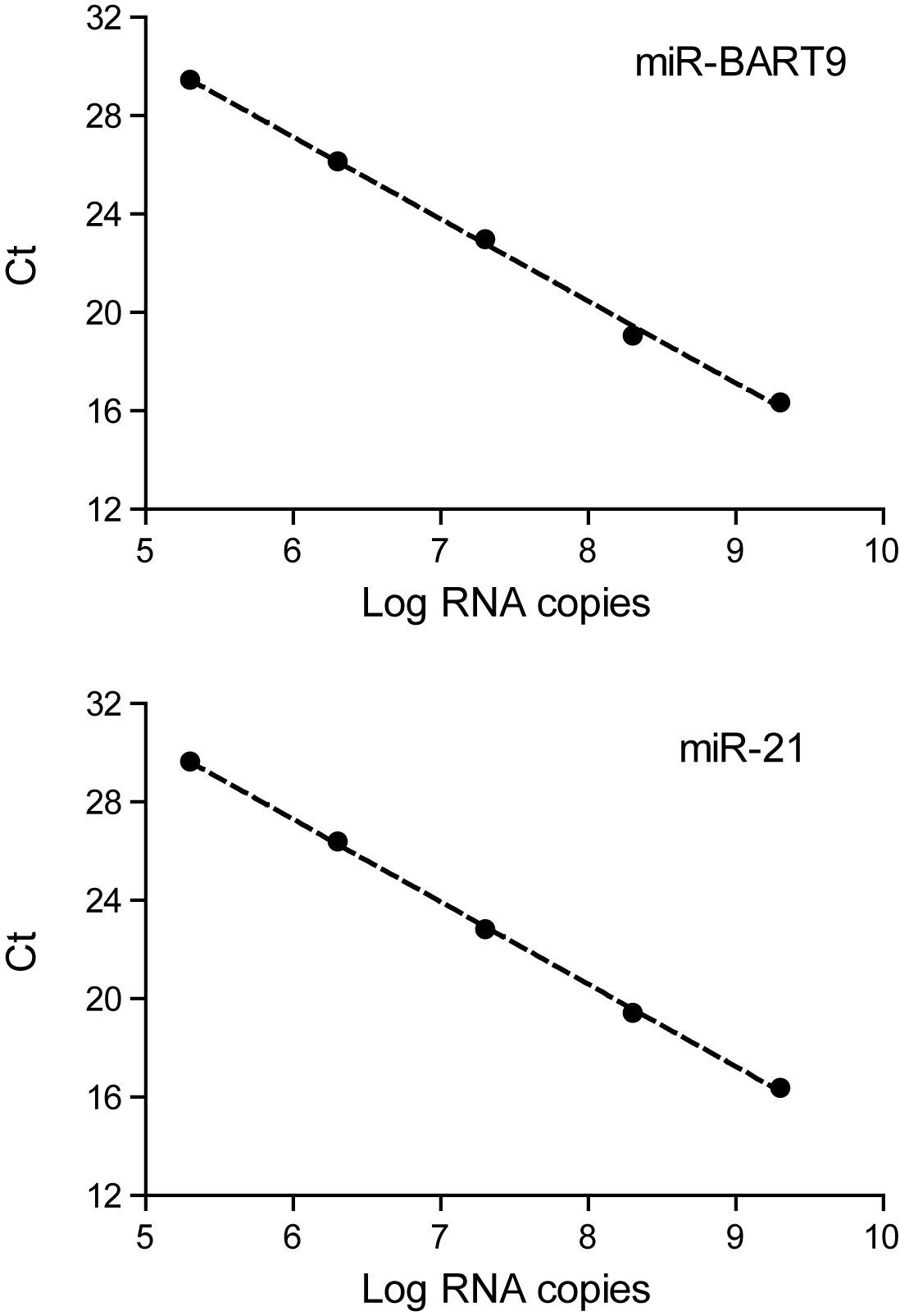

Supplement: Figure S1 — Standard curves for miR-BART9 and miR-21 qPCR assays. cDNA samples containing known copy numbers of synthetic miRNAs (3×10^5^ to 3×10^9^ copies) were used to test the absolute sensitivity of each qPCR assay. Standard curves obtained for miR-BART9 and miR-21 by plotting the cycle threshold (CT) values against log input RNA copy number are shown. (TIF) [file ppat.1003974.s001.tif]

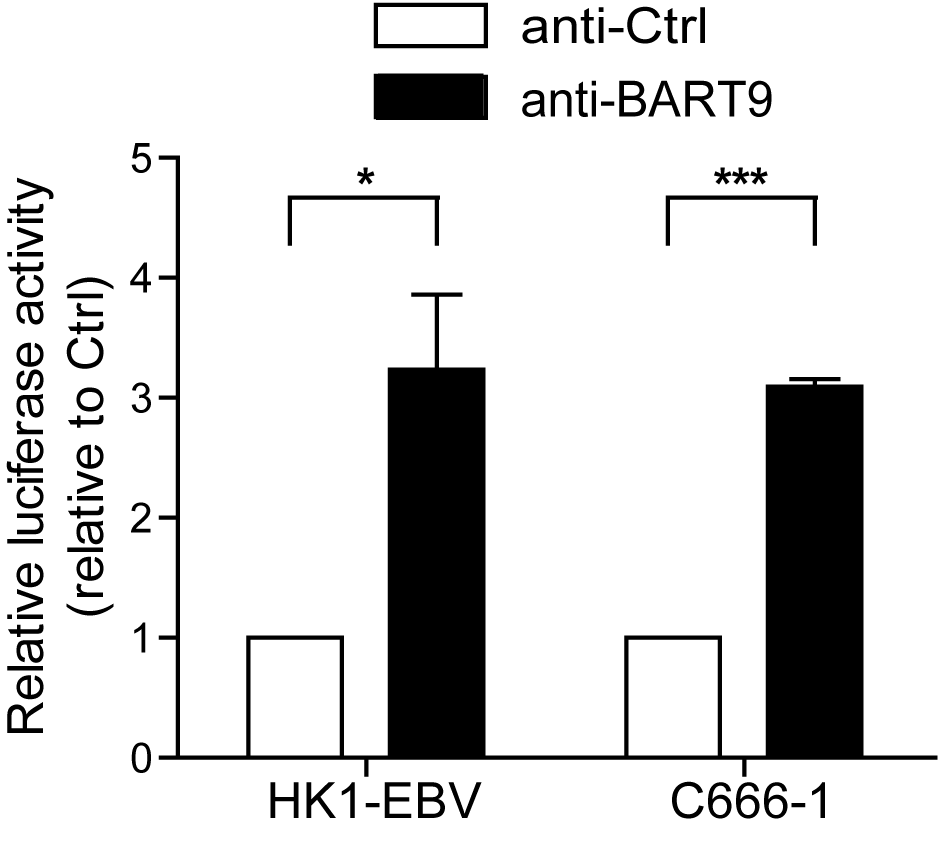

Supplement: Figure S2 — Determine the miR-BART9 activity after LNA treatment in EBV-positive NPC cells. To verify the knock-down efficiency of miR-BART9 in EBV-positive NPC cells, the miR-BART9 sensor luciferase reporter plasmid was used. Two copies of anti-sense miR-BART9 sequences which perfectly complementary to miR-BART9 were cloned into the pMIR-REPORT vector. HK1-EBV and C666-1 cells were co-transfected with a miR-BART9 sensor luciferase reporter vector and a 12.5 nM concentration of an LNA-modified miR-BART9 antisense oligo (anti-BART9) or a scramble control (anti-Ctrl) using Lipofectamine 2000 (Invitrogen). Cell lysates were harvested 48 hr after transfection and luciferase activity was measured by using the Dual-Luciferase Reporter Assay system (Promega). Luciferase activity was normalized to that of Renilla and calculated as a relative fold to control cells (anti-Ctrl). All data are presented as means values ± SEM of three independent experiments. *, P<0.05; ***, P<0.001. (TIF) [file ppat.1003974.s002.tif]

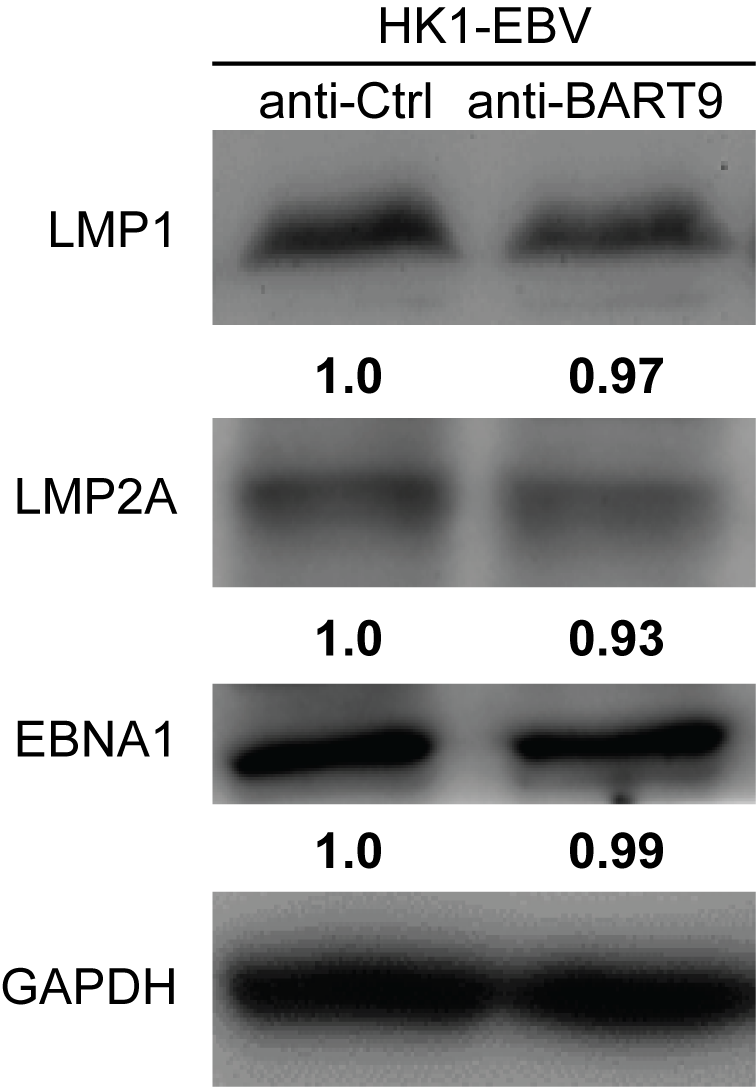

Supplement: Figure S3 — Depletion of miR-BART9 did not apparently affect LMP1, LMP2A or EBNA1 protein levels in HK1-EBV cells. HK1-EBV cells were treated with a 12.5 nM concentration of an LNA-modified miR-BART9 antisense oligo (anti-BART9) or a scramble control (anti-Ctrl) using Lipofectamine 2000 (Invitrogen). After 48 hours, cell lysates were harvested and performed western blots to determine the expression levels of LMP1, LMP2A and EBNA1. GAPDH protein was used as a protein loading control. LMP1, LMP2A and EBNA1 protein levels were normalized to GAPDH levels, and then compared with the anti-Ctrl cells whose normalized levels were expressed as 1.0. (TIF) [file ppat.1003974.s003.tif]

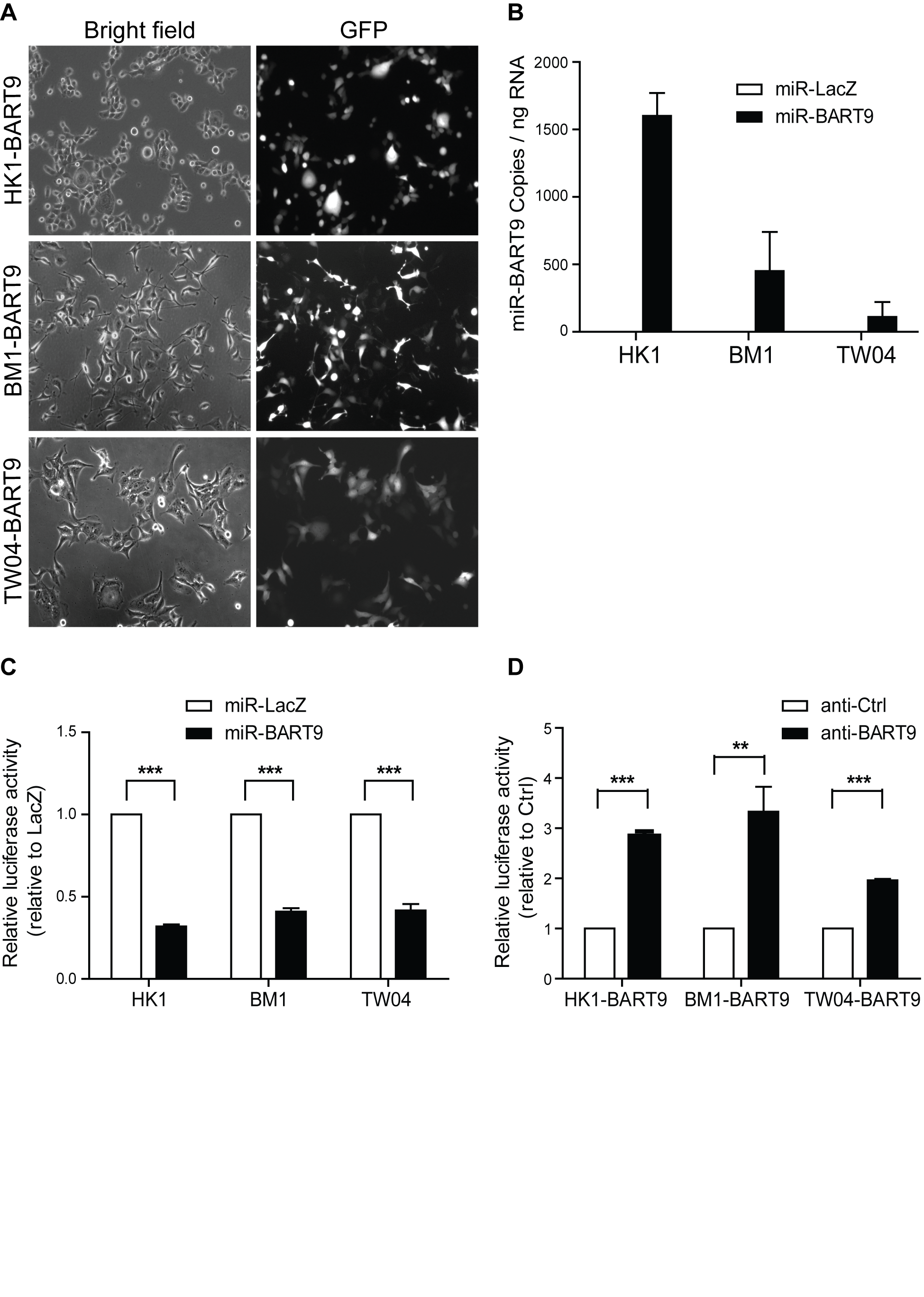

Supplement: Figure S4 — Expression levels of miR-BART9 in 3 EBV-negative NPC cell lines infected with lentivirus expressing the miR-BART9 or control (miR-LacZ) vector. (A) HK1, BM1 and TW04 NPC cells were transduced with lentivirus- expressing miR-BART9 vector, then photographed under an inverted fluorescence microscope (×100). The infection efficiency of lentivirus was over 90%. Importantly, no significant cell death was observed after virus infection. (B) After transduction with miR-BART9 LV and miR-LacZ in HK1, BM1 and TW04 NPC cells, miR-BART9 expression levels were detected by real-time RT-PCR analysis in the three cell lines. Error bars indicate standard deviations for four replicate assays. (C) Determine the miR-BART9 activity after infection with lentivirus expressing the miR-BART9 or control (miR-LacZ) vector in three EBV-negative NPC cell lines by luciferase reporter assay. (D) Determine the miR-BART9 activity after treating with a 12.5 nM concentration of an LNA-modified miR-BART9 antisense oligo (anti-BART9) or a scramble control (anti-Ctrl) in three miR-BART9 expressing NPC cells. Luciferase activity was normalized to that of Renilla and calculated as a relative fold to LacZ or control cells (anti-Ctrl). All data are presented as means values ± SEM of three independent experiments. **, P<0.01; ***, P<0.001. (TIF) [file ppat.1003974.s004.tif]

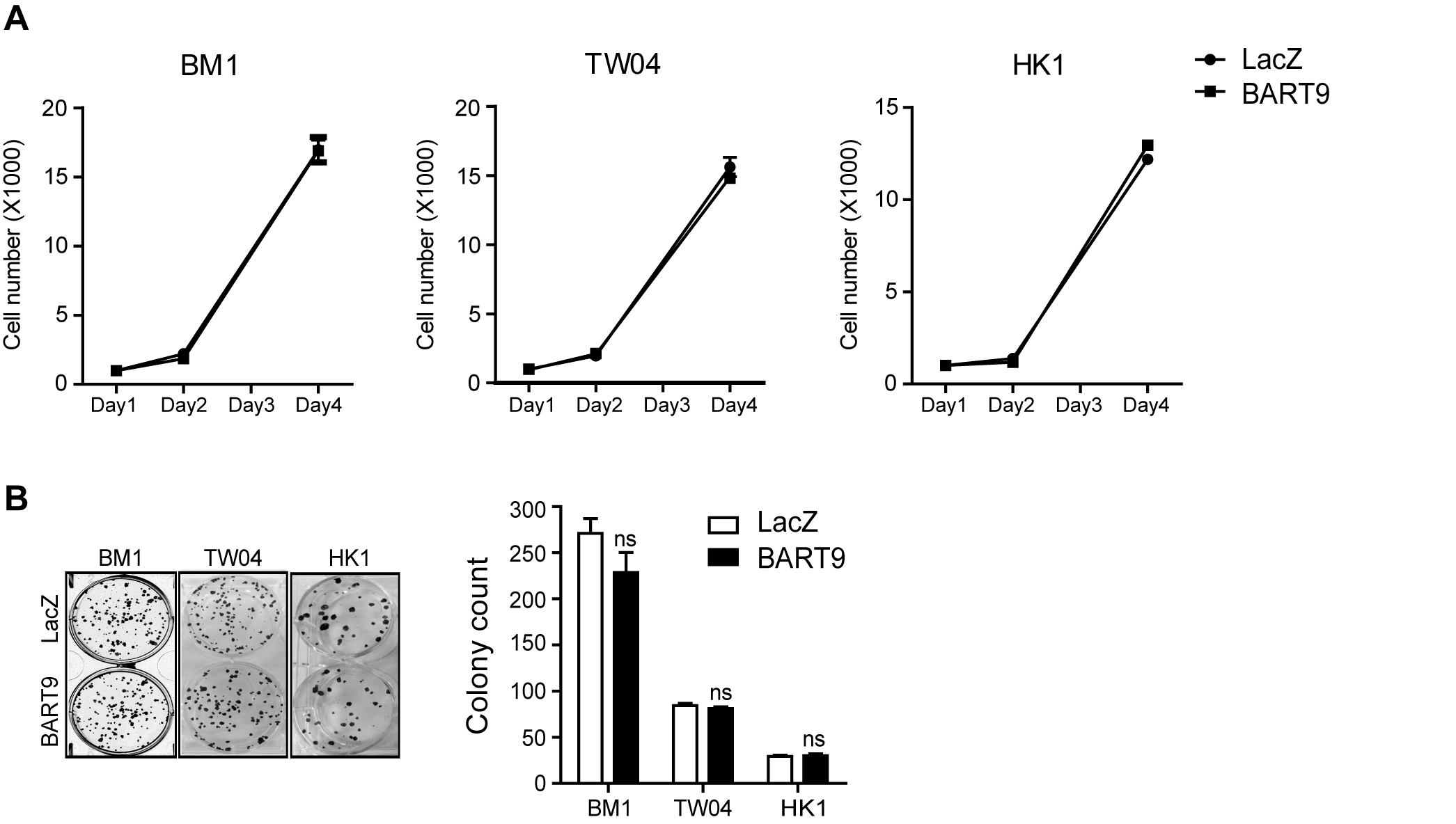

Supplement: Figure S5 — miR-BART9 has no significant effect on NPC cell growth in vitro . (A) BM1, TW04 and HK1 cells infected with lentivirus containing the miR-BART9 or control (LacZ) vector and miR-BART9 were plated in 96-well plates. Cells were fixed and stained with DAPI on days 1, 2 and 4. Cell numbers were determined using INCell 1000. The data were expressed as the mean cell counts ± SEM from six wells and a two-tailed Student's t-test was performed. (B) BM1, TW04 and HK1 cells infected with lentivirus containing the miR-BART9 or control (LacZ) vector and miR-BART9 were plated in 6-well plates. Colony formation activity was determined via crystal violet staining after 10 days in culture. The data are expressed as the mean colony counts ± SEM from three independent experiments and a two-tailed Student's t-test was performed. (TIF) [file ppat.1003974.s005.tif]

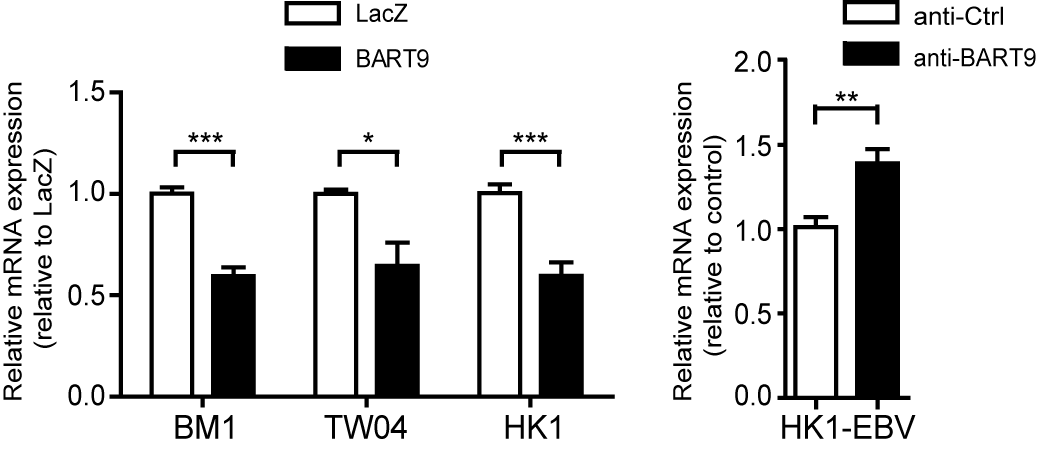

Supplement: Figure S6 — E-cadherin mRNA levels were decreased and increased in miR-BART9-overexpressing and knockdown NPC cells. Left panel: E-cadherin mRNA expression was measured with qRT-PCR in miR-BART9-overexpressing NPC cells. Right panel: Determine the mRNA level of E-cadherin in miR-BART9-depleting HK1-EBV cells. (TIF) [file ppat.1003974.s006.tif]

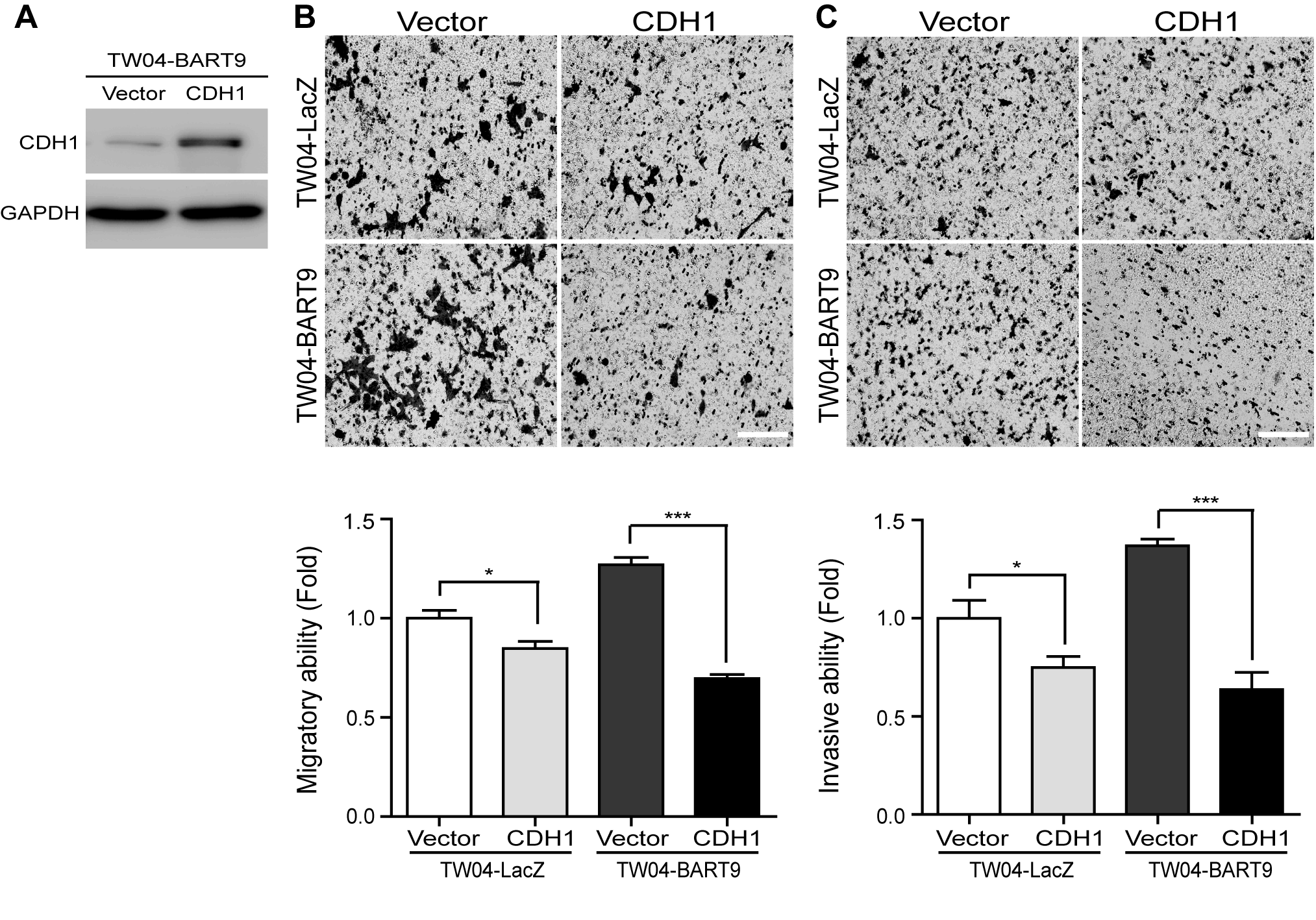

Supplement: Figure S7 — Rescue of E-cadherin reversed the effects of miR-BART9 on migration and invasion in NPC TW04 cells. (A) Rescue of E-cadherin expression in miR-BART9-expressing TW04 cells. Transwell migration assay (B) and Matrigel invasion assay (C) for miR-BART9- or LacZ-expressing TW04 cells, with or without ectopic expression of E-cadherin. Images of cells adhered to the lower surface of the filter insert from a representative experiment are shown. The numbers of migratory or invasive cells were quantified using image J and expressed as the fold change relative to the appropriate cell line (bar graphs). The data are expressed as the means ± SEM from three independent experiments and two-tailed Student's t-tests were performed (*, P<0.05; **, P<0.01; ***, P<0.001). Scale bar = 200 µm. (TIF) [file ppat.1003974.s007.tif]
