# Supplementary material for: The Epstein-Barr Virus-Encoded MicroRNA MiR-BART9 Promotes Tumor Metastasis by Targeting E-Cadherin in Nasopharyngeal Carcinoma
Source: PLoS Pathog. 2014 Feb 27;10(2):e1003974. doi: 10.1371/journal.ppat.1003974 (PMC3937311; doi:10.1371/journal.ppat.1003974)
Supplement: Table S1 — Synthetic RNA; LNA; Cloning primers. (PDF) [file ppat.1003974.s008.pdf]

Table S1 Synthetic RNA; Locked Nucleic Acid (LNA); Cloning primer

Gene		
ebv-miR-BART9-3p RNA	5'- rUrArA rCrArc rUrUrC rArUrG rGrGrU rCrCrC rGrUrA rGrU -3'	
hsa-miR-21-5p RNA	5'- rUrArG rCrUrU rArUrC rArGrA rCrUrG rArUrG rUrUrG rA -3'	
microRNA inhibitor control	5' - GTGTAACACGTCTATACGCCCA -3' (EXIQON; 199004-00)	
miR-BART9-3p LNA	5' -ACTACGGGACCCATGAAGTGTTA -3' (EXIQON; 136042-00)	
Pre-miR-BART9 cloning primer	Sense : TGCTGCAGCTGTTGTTTGTACTGGACCCTGAATTGGAAACAGTAACTTGGAT TCTGTAACACTTCATGGGTCCCGTAGTGACAACTATGCTG<br>Anti-sense : CCTGCAGCATAGTTGTCACTACGGGACCCATGAAGTGTTACAGAATCCAAG TTACTGTTTCCAATTCAGGGTCCAGTACAAACAACAGCTGC	
CDH1-3'UTR Mut	Forward :TTACTGTTTCTCAAGCA CTTTGG AGAAAAAAAT	Reverse :ATTTTTTTCTCCAAAGTGCTT GAGAAACAGTAA
miR-BART9 sensor	Forward :AGCTTAACACTTCATGG GTCCCGTAGTGATAACACTTCAT GGGTCCCGTAGT	Reverse :CTAGACTACGGGACCCATGA AGTGTTATCACTACGGGACCCATGAAG TGTTA
