# Supplementary material for: The Epstein-Barr Virus-Encoded MicroRNA MiR-BART9 Promotes Tumor Metastasis by Targeting E-Cadherin in Nasopharyngeal Carcinoma
Source: PLoS Pathog. 2014 Feb 27;10(2):e1003974. doi: 10.1371/journal.ppat.1003974 (PMC3937311; doi:10.1371/journal.ppat.1003974)
Supplement: Table S2 — Primers for qPCR analysis. (PDF) [file ppat.1003974.s009.pdf]

Table S2_Primers for quantitative RT-PCR analysis

Gene	Forward Primer (5'-3')	Reverse Primer (5'-3')
miR-BART9-3p	CGGCGGTAACACTTCATGGGTC	CTGGTGTCGTGGAGTCGGCAATTC
miR-21	CGGCGGTAGCTTATCAGACTGA	CTGGTGTCGTGGAGTCGGCAATTC
miR-93	CGGCGGAAAGTGCTGTTCGTGC	CTGGTGTCGTGGAGTCGGCAATTC
U6	CTCGCTTCGGCAGCACA	AACGCTTCACGAATTTGCGT
LMP1	GTCCTCTATTCCTTTGCTCTCATG	TGCCTGTCCGTGCAAATTC
LMP2A	CCGTCACTCGGACTATCAAC	TGAGATGAGTCATCCCGTGGA
EBNA1	AGGATGCGATTAAGGACCTTGTT	CCATCGTCAAAGCTGCACAC
MMP1	GATGGACCTGGAGGAAATC	GTCCAAGAGAATGGCCGA
MMP2	CTGGAGAACTAGAGAAGGACC	CCTGGGAGGAGTACAGTCA
MMP9	CCACTGCTGGCCCTTCTA	CCCTGCCCTCAGAGAAT
MMP10	CTAGACTAATAGCTGATGACTTTCC	TGTTACTCTTTAATATGTGTGTCACC
MMP12	ATGGACCCTGGTTATCCC	ACGTTGGAGTAGGAAGTCA
CDH1	CTGAAGTGACTCGTAACGAC	ACGAGCAGAGAATCATAAGG
CTNNA1	AGTCACTGTTCGTCACTCA	ACAGCTAGAAGTAGGCTCC
Vimentin	ACATTGAGATTGCCACCTAC	AACCGTCTTAATCAGAAGTGTC
EEF1A1	CAATGTGGGCTTCAATGTCAA	CATAGCCGGCGCTTATTTG
