# Supplementary material for: The Epstein-Barr Virus-Encoded MicroRNA MiR-BART9 Promotes Tumor Metastasis by Targeting E-Cadherin in Nasopharyngeal Carcinoma
Source: PLoS Pathog. 2014 Feb 27;10(2):e1003974. doi: 10.1371/journal.ppat.1003974 (PMC3937311; doi:10.1371/journal.ppat.1003974)
Supplement: Table S3 — Enrichment analysis of predicted miR-BART9 targets in KEGG cell signaling pathway database. (PDF) [file ppat.1003974.s010.pdf]

Table S3_Enrichment analysis of predicted miR-BART9 targets in KEGG cell signaling pathway database

Pathway	Count	P_value	Enrichment
Small cell lung cancer	17	7.70E-03	2
Pathways in cancer	47	9.40E-03	1.4
Pathogenic Escherichia coli infection	12	2.30E-02	2.1
Focal adhesion	30	2.50E-02	1.5
Amyotrophic lateral sclerosis (ALS)	11	3.40E-02	2.1
p53 signaling pathway	13	3.40E-02	1.9
ECM-receptor interaction	15	3.60E-02	1.8
Cell cycle	20	3.90E-02	1.6
Regulation of actin cytoskeleton	30	5.30E-02	1.4
